# Anterior-posterior patterning in the chaetognath *Spadella cephaloptera* informs bilaterian nervous system and tail evolution

**DOI:** 10.1038/s42003-025-09398-6

**Published:** 2025-12-26

**Authors:** June F. Ordoñez, Tim Wollesen

**Affiliations:** 1 Unit for Integrative Zoology, Department of Evolutionary Biology, University of Vienna, Vienna, Austria; 2 Vienna Doctoral School of Ecology and Evolution (VDSEE), University of Vienna, Vienna, Austria

**Keywords:** Zoology, Molecular biology

## Abstract

The formation of the bilaterian anterior–posterior axis relies on deeply conserved patterning systems, yet how these evolved remains incompletely understood. As sister to or within Gnathifera, chaetognaths provide an informative phylogenetic context for investigating anterior–posterior patterning, yet their developmental genetics remain poorly studied. Here, we examine anterior patterning (*otx, nk2.1, six3/6*) and Hox gene expression in the chaetognath *Spadella cephaloptera*. Anterior patterning genes are expressed in cerebral neural regions as in other bilaterians. In contrast, Hox genes, including the previously undescribed *med6*, *postC*, and *postD*, show staggered expression along the nervous system, resembling the proposed ancestral bilaterian condition. Posterior Hox genes are also expressed in the postanal tail, a trait only present in chaetognaths and chordates, suggesting a link between Hox expansion and the independent emergence of this structure. Our results indicate that chaetognaths retain ancestral bilaterian features and provide insights into Hox-driven lineage-specific innovations within Spiralia.

## Abstract (web summary)

Expression of anterior patterning and Hox genes in the chaetognath *Spadella cephaloptera* reveals conserved features of bilaterian body patterning and offers insight into how Hox genes contributed to nervous system and tail evolution.

## Introduction

The anterior–posterior (AP) axis is a fundamental organizational feature of bilaterian body plans, established by conserved transcription factors that establish regional identity during development. Anterior-specific genes (e.g., *otx*, *nk2.1*, *six3/6, foxQ2*) specify anterior neural and head structures, while Hox genes control positional identities along the AP axis (e.g.^1–4^). In many bilaterians, these genes also regulate neural regionalization through staggered expression in the developing central nervous system (CNS), indicating a conserved mechanism of CNS patterning across lineages^5–9^. While these mechanisms are well described in model systems such as vertebrates, arthropods, and annelids, our understanding of how these genes contribute to AP axis formation across the broader diversity of bilaterians remains incomplete.

Spiralians have historically been underrepresented in studies of Hox gene expression. However, recent research on annelids, molluscs, and other spiralian lineages has begun to uncover a variety of Hox expression profiles. Several lophotrochozoans, including some annelids and molluscs, exhibit canonical staggered Hox expression along the AP axis, particularly in the developing nervous system and non-neural ectoderm^10–14^. Others, however, display more restricted or non-staggered expression profiles, with Hox gene expression confined to particular tissues or developmental stages^15–19^. This variability highlights both conserved and divergent aspects of Hox function across Spiralia, but drawing broader evolutionary conclusions remains difficult due to limited taxon sampling, especially among underrepresented but phylogenetically informative lineages that appear to have maintained aspects of the ancestral body plan.

Chaetognaths (arrow worms) offer a particularly compelling model for comparative developmental studies. These marine invertebrates occupy a contentious phylogenetic position due to their mosaic of morphological and molecular traits, some of which resemble those of deuterostomes, others of protostomes, or suggest they form a distinct lineage altogether^20–35^. Recent phylogenomic analyses suggest that chaetognaths are either nested within Gnathifera or as a sister lineage to the group^24,27^. This phylogenetic positioning offers a unique opportunity to infer ancestral spiralian features and their roles in the evolution of bilaterian body plans.

Chaetognaths share several developmental and morphological features with rotifers (Monogononta, Bdelloidea, and Seisonidea), including the absence of spiral cleavage, the specification of primordial germ cells (PGCs) through preformation, a chitinous feeding apparatus, a trunk without external motile cilia, a postanal structure (tail/foot), and the central-post class Hox gene, *medpost*^16^. These shared traits support the proposed evolutionary link between chaetognaths and gnathiferans. Despite this phylogenetic proximity, chaetognaths retain a full complement of anterior, central, and posterior Hox genes^31,36^, in contrast to rotifers, which have a highly reduced Hox repertoire and entirely lack posterior-class genes^16,37^. Nevertheless, a comprehensive understanding of AP axis patterning in chaetognaths remains limited compared to other spiralians. Earlier chaetognath studies have examined the Hox complement of *Spadella cephaloptera* and *Flaccisagitta enflata*^31,36^, revealing a repertoire, although incomplete, that broadly resembles that of other bilaterians. Papillon et al.^38^ first reported expression of a central-class Hox gene in the ventral nerve center (VNC), suggesting a possible neural role. By comparison, in the rotifer *Brachionus manjavacas*, Hox gene expression is restricted to developing neural tissues and appears only partially staggered^16^. For Gnathifera, expression of anterior patterning genes *nk2.1* and *pax6* has been described in the brain of the juvenile rotifer *Epiphanes senta*^39^, and *pax6* is also expressed in the anterolateral region of the rotifer *Brachionus plicatilis* during embryogenesis, although the exact tissue identity remains uncertain^40^. These differences, combined with limited data available, underscore the need for comparative developmental studies in chaetognaths to clarify whether AP patterning in this lineage reflects conserved spiralian and/or bilaterian mechanisms, or represents a distinct innovation within chaetognaths.

While this manuscript was under revision and already uploaded to BioRxiv^41^, a chromosome-level genome of *Paraspadella gotoi* was published, revealing an expanded, physically linked Hox cluster shaped by local duplication and partially staggered expression for a subset of genes in the early juvenile VNC^42^. This study provides a valuable genomic context for chaetognath Hox organization but leaves expression coverage incomplete. Here, we examine the expression profiles of the AP-patterning genes *otx*, *nk2.1*, and *six3/6* in the chaetognath *S. cephaloptera* during embryonic and early post-hatch development, providing the most extensive spatiotemporal data to date on anterior-patterning genes (*otx*, *nk2.1*, *six3/6*) and broad coverage of the Hox repertoire in a chaetognath. We show that AP-patterning genes are expressed in the cerebral ganglion and developing head, consistent with their conserved role in brain and anterior tissues specification across Bilateria. We also describe an updated Hox gene complement for *S. cephaloptera*, including several previously undescribed members, and demonstrate staggered Hox expression along the AP axis of the VNC and postanal tail. These spatially organized expression patterns suggest that chaetognaths retain core bilaterian mechanisms of CNS patterning, which appear to have been secondarily lost in several other bilaterian lineages, despite their peculiar developmental traits and uncertain phylogenetic position.

## Methods

### Identification of gene homologs

We retrieved AP patterning (*otx*, *six1/3*, *nk2.1*, Hox) genes from the *S. cephaloptera* draft transcriptome^43,44^ using blastx searches^45^ against protein sequences from the NCBI GenBank non-redundant protein database. We also searched for *gastrulation brain homeobox* (*gbx*) and *forkhead homeobox Q2* (*foxQ2*), two transcription factors involved in axial patterning and neural development in many bilaterians^3,13,14^, but found no *gbx* and *foxQ2* homologs in the *S. cephaloptera* transcriptome. To determine gene orthology, phylogenetic analyses were conducted using reference protein sequences from a broad range of bilaterians (Supplementary Table 1). Additionally, Hox genes from selected chaetognath species, inferred from transcriptomic data, were also incorporated in the phylogenetic reconstruction. Briefly, raw RNA-seq reads from several chaetognath species generated by Marlétaz et al.^27^ were downloaded from the GenBank Sequence Read Archive. Raw reads of the rotifer *Rotaria rotatoria* were also included. Adapter removal, quality filtering, and trimming were performed using Trimmomatic (v0.39)^46^, and transcriptome assemblies were generated using rnaSPAdes (v4.1)^47^, both with default parameters. The resulting transcriptomes were screened for Hox genes using DIAMOND blastx (v2.1.11)^48^ against a custom database of protostome Hox gene sequences used in this study, applying an e-value threshold of 1e-^20^. SRA accession numbers used in the analysis are provided in Supplementary Table 2 and the resulting Hox sequences from the assemblies are presented in Supplementary Note.

Amino acid sequences were aligned using MAFFT (v7.490)^49,50^, implemented in Geneious Prime (v2023.0.1). The resulting multi-sequence alignments were then trimmed with ClipKit (v2.2.3)^51^. Maximum likelihood (ML) analyses were performed in IQTREE (v2.3.6), incorporating ModelFinder for amino-acid substitution model selection and conducting 1000 ultrafast bootstrap replicates^52–54^, along with SH-aLRT test replicates for Hox genes^55^. Substitution models used in phylogenetic analyses are indicated in Supplementary Table 2. Additionally, phylogenetic reconstruction for Hox genes was also performed using Bayesian inference in MrBayes (v3.2.7a)^56^, employing the same substitution model as the ML analysis (i.e., VT + G + F). The analysis was run for 50 million generations, sampling every 1000 generations across eight independent runs, each with four chains. Convergence was assessed using multiple diagnostics in MrBayes: Average Standard Deviation of Split Frequencies (ASDSF), Potential Scale Reduction Factor (PSRF), and Effective Sample Size (ESS). While ASDSF of 0.0206 is slightly above the recommended 0.01 threshold, ESS values exceeded 500 across parameters, and PSRF remained approximately 1.000 for all numeric parameters. This follows standard recommendations for Bayesian phylogenetic inference indicating robust convergence despite a minor ASDSF deviation^57,58^. The first 25% of sampled generations were discarded as burn-in prior to calculating the consensus tree.

### Animal collection

Live adult specimens of *S. cephaloptera* (Busch, 1851) were collected in June 2024 during low tide from the intertidal zone off Roscoff, France (48°43'47.2“N 3°59'12.2“W). Specimens were obtained by sweeping hand nets and plankton nets through the shallow algal meadows and were subsequently housed in aquaria in the aquatic research facility of the University of Vienna. The aquaria contain a combination of natural and artificial seawater, with a temperature and salinity of 14°C and 35‰, respectively. Sexually matured individuals were transferred to plastic Petri dishes (14.1 cm radius), which were refreshed with natural sea water every day. The animals were fed with *Artemia sp*. nauplii until they laid eggs. Embryos and hatchlings (8–20 h post-hatching) were manually collected. Additionally, some hatchlings were allowed to develop to early juveniles (7–10 days post-hatching) before they were sampled.

All specimens were fixed in 4% paraformaldehyde in a buffer containing 0.1 M MOPS pH 7.4, 2 mM EGTA, 1 mM MgSO_4,_ 2.5 M NaCl. The specimens used for whole-mount RNA Fluorescence in situ Hybridization (RNA-FISH) were fixed overnight (14–18 h) at 4 °C, while for whole-mount Hybridization Chain Reaction (HCR-FISH)^59–61^, they were fixed for only 1–1.5 h. Following fixation, the specimens were washed thrice for five minutes each in PBTw (1x PBS, pH 7.5 with 0.1% Tween-20), washed three times in ice-cold methanol for 10 minutes each, and then stored in 100% methanol at −20°C until use.

### Sequencing, riboprobe synthesis, and whole-mount fluorescent in situ hybridization (FISH)

RNA extracted from hatchlings and adults using RNAqueous™-Micro Total RNA Isolation Kit (Invitrogen GmbH, Karlsruhe, Germany) was used to generate a cDNA library with First Strand cDNA Synthesis Kit for RT-PCR (AMV) (Roche Molecular Biochemicals, Vienna, Austria). Riboprobes were generated either directly from PCR amplicons or from plasmid inserts. For PCR-derived riboprobes, reverse primers carried a 5’ T7 promoter sequence and resulting products were purified with QIAquick PCR Purification Kit (Qiagen Vertriebs GmbH, Vienna, Austria). For cloning, gene fragments amplified with PCR were cloned into pGEM-T Easy vectors (Promega, Mannheim, Germany), transformed into competent *Escherichia coli* cells, and then purified with QIAprep® Miniprep (Qiagen Vertriebs GmbH, Vienna, Austria). The purified fragments from both methods were sent to Microsynth Austria for Sanger sequencing to validate their specificity. Primer sequences and PCR conditions are listed in Supplementary Table 2. Digoxigenin (DIG)-labeled sense riboprobes from the PCR product and linearized DNA were generated using the T7 polymerase and digoxigenin RNA labeling mix kit (Roche Diagnostics, Mannheim, Germany). Total riboprobe concentrations were estimated using NanoDrop 2000 spectrophotometer (ThermoFisher Scientific, Massachusetts, USA). Traditional whole-amount fluorescent in situ hybridization (RNA-FISH) experiments on hatchlings were conducted following the procedure described in refs. ^43,62^. HCR-FISH^59,63^ was carried out with genes (*Sce-otx, Sce-nk2.1, Sce-hox2*, *Sce-medpost*, *Sce-postB*, *Sce-postC*, and *Sce-postD*) for which riboprobes failed to work with RNA-FISH. These probes were also used in embryos, as RNA-FISH is not effective with early embryonic stages^62^. HCR Probes for each gene were designed using the program *insitu_probe_generator.py* (https://github.com/rwnull/insitu_probe_generator^64^) and were purchased from Integrated DNA Technologies (München, Germany). Hairpins were purchased from Molecular Instruments, Inc. (California, USA). HCR-FISH was performed following Choi et al.^59^, with additional modifications from Bruce et al.^65^. Further adjustments were made for embryos, with dextran sulfate concentration reduced from 10% to 2% to minimize severe morphological distortion. Information on the PCR primer sequences and HCR probes is provided in Supplementary Table 3 and Supplementary Data 1, respectively. For hatchlings and juveniles, 2–3 individuals were analyzed per run across two independent runs (total *n* = 4–6 per stage). For embryos, 1–2 individuals were analyzed per run across two independent runs (total *n* = 2–4). An expression pattern was considered supported if it was observed in ≥3 hatchling/juvenile individuals and in ≥2 embryos.

Post-embryonic stages were incubated in Vectashield® (Vector Laboratories, California, USA) for 20 minutes before mounting with 70% 2,2’-thiodiethanol (TDE) in PBS. To minimize embryo deformation, specimens were incubated in a series of Vectashield®-PBS solutions (10%, 30%, 60%, 80%, and 100%) for 8 minutes at each concentration. Clearing was then achieved by titrating 100% TDE into 2 µL Vectashield containing the embryo in 0.5 µL increments at 5-min intervals (6–8 additions) until optically cleared. The specimens were then mounted in the solution used for documentation. Specimens were scanned with Leica TCS SP5 (Leica Microsystems, Heidelberg, Germany) confocal laser scanning microscope. Brightness/contrast adjustments and Z-projections were performed with Fiji^66^. Figure assembly and schematic drawings were prepared in Inkscape (https://inkscape.org).

### Reporting summary

Further information on research design is available in the Nature Portfolio Reporting Summary linked to this article.

## Results

### Staging overview for *Spadella cephaloptera* embryogenesis

**Fig. 1:**
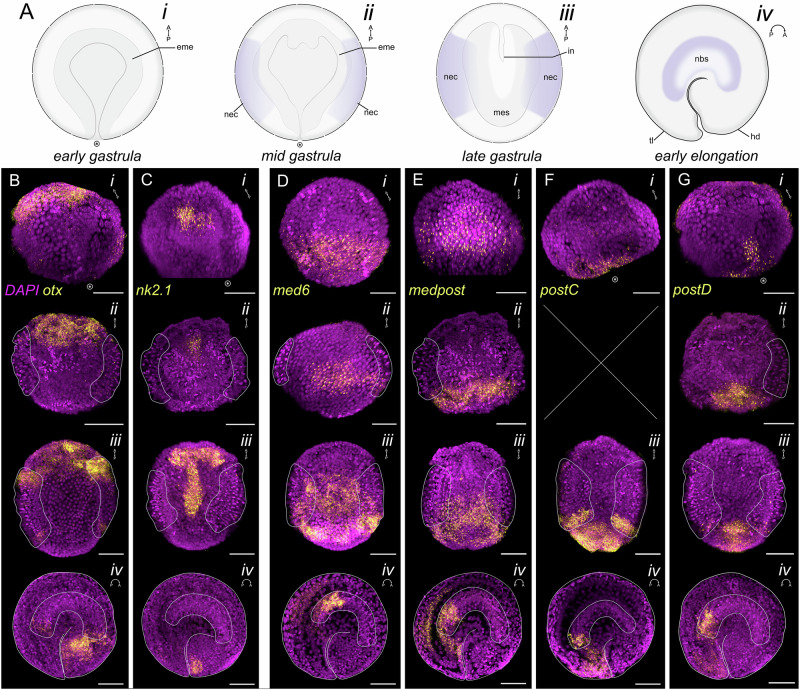
Expression patterns of anterior patterning and Hox genes in *Spadella cephaloptera* embryos. Gene transcripts (yellow) are visualized with AlexaFluor-647 and cell nuclei with DAPI (purple). **A** Schematic representations of early (*i*), mid (*ii*), and late gastrula (*iii*) in ventral view, and early elongation stage (*iv*) in lateral view. **B**–**G** Expression patterns of *Sce-otx* (**B**), *Sce-nk2.1* (**C**), *Sce-med6* (**D**), *Sce-medpost* (**E**), *Sce-postC* (**F**), *Sce-postD* (**G**) across embryonic stages (*i* – *iv*). No expression data are available for *Sce-postC* at the mid gastrula stage. Purple outlines demarcate the neuroectoderm (*ii*, *iii*) and the developing ventral nerve center (*iv*). Scale bars: 50 μm. Encircled asterisk indicates the position of the blastopore. Orientation is indicated in the upper right corner of each panel. ect, ectoderm; eme, endomesoderm; hd, head bud; in, intestine; mes, mesoderm; nbs, neuroblast of the developing VNC; nec, neuroectoderm; tl, tail bud.

Embryonic stages of *S. cephaloptera* were defined by morphology following John^67^, rather than time post-fertilization, because oviposition timing under laboratory conditions was unreliable (Fig. 1Ai-iv; Supplementary Fig. 1Ai–iv). Early gastrula: visible blastopore; no neuroectodermal thickening (Supplementary Fig. 1Ai). Mid gastrula: ventrolateral neuroectoderm emerges as a mitotically active thickening (Supplementary Fig. 1Aii). Late gastrula: axial elongation, intestinal invagination, blastopore closure, and further internalization of neuroectodermal cells (Supplementary Fig. 1Aiii). Early elongation: curvature along the AP axis, distinct head and tail buds, and aggregation of the neuroectodermal cells at the medioventral midline forming the nascent VNC (Supplementary Fig. 1Aiv). These landmarks frame the spatiotemporal gene expression analyses in the succeeding sections.

### Gene orthology and expression patterns of anterior-patterning genes

We identified single-copy gene orthologs of *otx, nk2.1*, and *six3/6* from the *S. cephaloptera* draft transcriptome by BLAST and ML phylogeny (Supplementary Figs. 2 and 3; see Ordoñez and Wollesen^62^ for *Sce-nk2.1* orthology assignment).

#### Otx expression

In the early gastrula, *Sce-otx* is expressed in the anterior ectoderm and PGCs (Fig. 1Bi; Supplementary Fig. 1Bi). At the mid-gastrula stage, expression persists in the anterior ectoderm (Fig. 1Bii; Supplementary Fig. 1Bii). By the late gastrula stage, it extends to both the anterior and posterior regions of the neuroectoderm (Fig. 1Biii; Supplementary Fig. 1Biii). During early elongation, *Sce-otx* shows broad expression in the head bud and the polar tips of the lateral neuroblasts of the nascent VNC (Fig. 1Biv; Supplementary Fig. 1Biv).

**Fig. 2:**
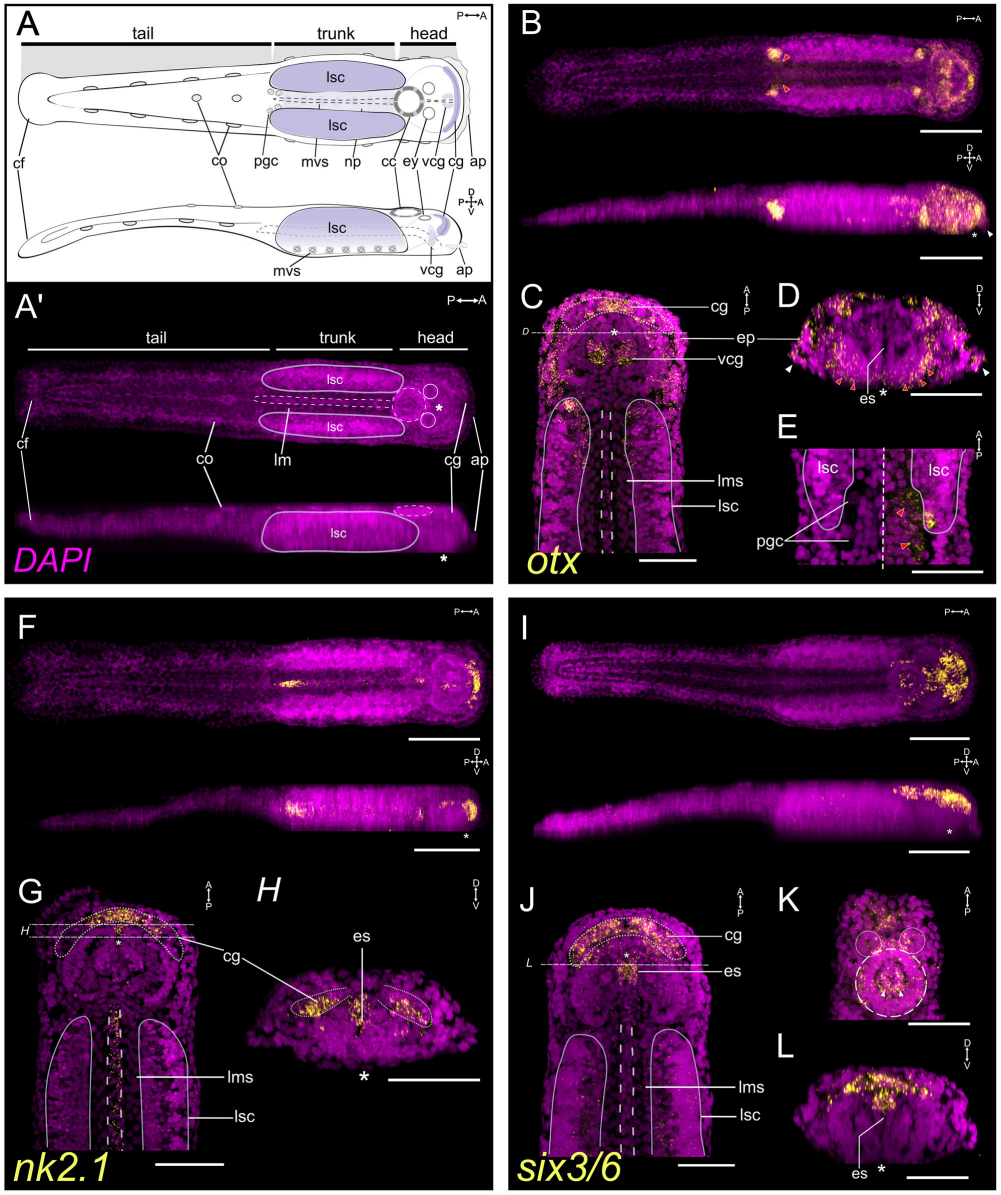
Expression patterns of anterior patterning genes in the hatchling (1 dph) of *Spadella cephaloptera*. Gene transcripts (yellow) are visualized with AlexaFluor-647 (**B**–**H**) or AP-Fast Blue (**I**–**L**), and cell nuclei are counterstained with DAPI (purple). **A**, **A**’ General morphology of a hatchling shown in schematic drawing (**A**) and maximum intensity projection of DAPI-stained whole animal (**A**’). Top: dorsal view; bottom: lateral view. **B**–**E** *Sce-otx* expression pattern. **B** Dorsal and lateral maximum projections. **C** Dorsal section of the anterior body showing expression in the mid-anterior cerebral ganglion (cg) and presumptive ventral cephalic ganglia precursors (*vcg*). **D** Transverse section of the anterior head showing *Sce-otx* expression at the base of the head (red arrowheads) and in the cephalic adhesive papillae (*ap*, white arrowheads). **E** Higher magnification of primordial germ cells (*pgc*; red arrowheads), shown in DAPI only (left) and merged channels (right). **F**–**H** *Sce-nk2.1* expression pattern. **F** Dorsal and lateral maximum projections. **G** Dorsal section of the anterior body showing expression in the cerebral ganglion and intestine (outlined). **H** Transverse section posterior to the cerebral ganglion showing expression in the anterior esophagus (*es*). **I**–**L** *Sce-six3/6* expression pattern. **I** Dorsal and lateral maximum projections. **J**, **L** Dorsal sections of the anterior body showing expression in the cerebral ganglion and dorsal esophagus. **K** Dorsal section along the dorsal-most portion of the head showing *Sce-six3/6*+ cells in the eyes (solid circles) and inner corona ciliata (dashed circle). **L** Transverse section through the head along the esophagus, showing *Sce-six3/6* expression in its dorsal domain. Scale bars: 50 μm, except panels *B*, *F*, and *I* (100 μm). Asterisk indicates the position of the mouth opening. Orientation is indicated in the top-right corner of each panel. ap cephalic adhesive papillae, cc corona cilata, cf caudal fin, cg cerebral ganglion, co ciliary tuft/fence organ, es esophagus, ey eye, in intestine, lms longitudinal muscle somata, lsc lateral somata clusters, mvs medioventral somata clusters, np neuropil of the ventral nerve center, pgc primordial germ cells, vcg ventral cephalic ganglia precursor.

In hatchlings, *Sce-otx* is broadly expressed in the head, with a strong signal concentrated in the anterior cerebral ganglion (Fig. 2B), presumptive ventral cephalic ganglia precursors (Fig. 2C), head epidermis (Fig. 2C–E), cephalic adhesive papillae (white arrowheads in Fig. 2B, D), and around the future mouth opening (asterisk in Fig. 2B, D). In the VNC, *Sce-otx* expression remains in the anterior and posterior tips of the lateral somata clusters and shows a bilateral streak pattern along the anterior inner cells (Fig. 2B, C). Early juveniles express *Sce-otx* throughout the head (Supplementary Fig. 4B–F), including the brain (dotted outline in Supplementary Fig. 4C), corona ciliata (dashed circle in Supplementary Fig. 4C), eyes (circles in Supplementary Fig. 4C), perioral epidermis (red arrowheads in Supplementary Fig. 4D, E), hood (Supplementary Fig. 4E), and cephalic epidermis (Supplementary Fig. 4B, C, E). In the trunk, *Sce-otx* is expressed in the lateral somata clusters (Supplementary Fig. 4B, D) and in the proliferating PGCs (red arrowheads in Supplementary Fig. 4F). PGC expression is detected at all stages except late gastrula and early elongation (Fig. 2E; red arrowheads in Supplementary Fig. 1Bi, ii and Supplementary Fig. 4F).

#### Nk2.1 expression

*Sce-nk2.1* expression is concentrated in the mid anterior cell population of the endomesoderm in early and mid gastrula (Fig. 1Ci, ii; Supplementary Fig. 1Ci, ii). In the late gastrula, these *Sce-nk2.1*+ cells extend posteriorly along the midline, a domain that will give rise to the future intestine (Fig. 1Ciii; Supplementary Fig. 1Ciii). A distinct anteroventral ectodermal domain also emerges, though its identity remains uncertain (inset *iii’* in Supplementary Fig. 1Ciii). During early elongation stage, *Sce-nk2.1* signal is detected in the mid-anterior head bud and in cells located in the developing tail, here referred to as tail bud (white arrowheads in Supplementary Fig. 1Civ).

At hatching, *Sce-nk2.1* expression appears as a semi-arch, marking the anterior part of the developing brain (Fig. 2F – H) and outlines the digestive track, from the esophagus to the posterior gut, with the strongest signal intensity in the hindgut (Fig. 2F). In early juveniles, expression is observed in the anterior tip of the brain and in patches of neurons in its posterior domain (Supplementary Fig. 4G, H). *Sce-nk2.1* remains active in the cells of the intestine at this stage (Supplementary Fig. 4G, I, J).

#### Six3/6 expression

*Sce-six3/6* is mostly expressed in the head of hatchlings (Fig. 2I - L), including the cerebral ganglion, eyes, corona ciliata, anterodorsal epidermal cells, and the anterodorsal cells of the esophagus (Fig. 2J, L). The lateral somata clusters show a faint *Sce-six3/6* expression (Fig. 2J). *Sce-six3/6* expression in early juveniles is observed in the brain (Supplementary Fig. 4K, dotted outline in L), corona ciliata (dashed outline in Supplementary File 4 Fig. L), eyes (circles in Supplementary Fig. 4L), vestibular ganglia, esophageal ganglia (Supplementary Fig. 4M), and in the lateral somata clusters (Supplementary Fig. 4K, N).

#### The *Spadella* Hox gene complement

**Fig. 3:**
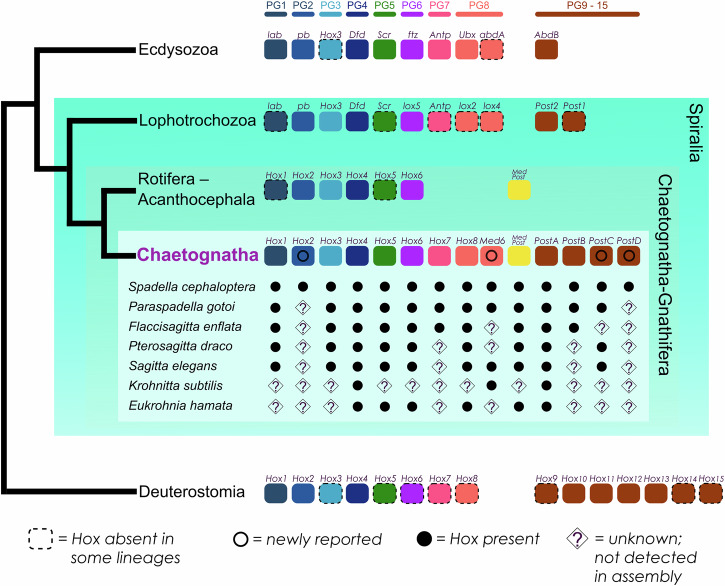
Comparative overview of Hox gene complements across major bilaterian lineages. A schematic phylogeny based on Marlétaz et al.^27^ illustrates the generalized Hox gene complement for Deuterostomia, Ecdysozoa, Lophotrochozoa, and Rotifera–Acanthocephala, alongside a species-level comparison within Chaetognatha. Boxes to the right of each group or species indicate the presence or undetected status of Hox genes across paralog groups (PG1–PG8 and posterior Hox genes, where applicable). Filled circles indicate identified Hox genes. Diamonds with question marks denote genes not retrieved from transcriptomic data in chaetognath species, though absence cannot be confirmed due to limitations such as low sequencing coverage or stage-specific expression.

Chaetognaths are currently known to possess ten Hox genes representing five paralogous groups: the anterior class Hox gene *hox1* (PG 1), *hox3* (PG 3), the central class genes *hox4*–*hox8 (*PG 4-8), the central/posterior chimera *medpost*, and the posterior class genes *postA* and *postB* (PG 9-15)^31,36^. From the *S. cephaloptera* draft transcriptome, we recovered orthologs of previously reported Hox genes in chaetognaths using ML (Supplementary Fig. 5) and Bayesian phylogenetic analyses (Supplementary Fig. 6), while also identifying four previously undescribed Hox genes in chaetognaths: *Sce-hox2*, *Sce-med6*, *Sce-postC*, and *Sce-postD*. Surveying public chaetognath datasets reveal *med6* and *postC* in multiple species (e.g. *Paraspadella gotoi* and *Sagitta elegans*), while *hox2* and *postD* were not recovered (Fig. 3). Given that the majority of chaetognath Hox genes have already been characterized in earlier studies^31,36^, we focus here on the four newly identified genes recovered from our transcriptome survey, which expand the known Hox complement in this group.

Orthology analysis assigns *Sce-hox2* to the *hox2*/*proboscipedia* (*pb*) cluster, where it contains five amino-acid residues conserved in PG2: R (position 2), L (4), N (10 and 23), and V (45) (Supplementary Fig. 7). All *med6* sequences form a well-supported clade with chaetognath *hox8*, both nested within PG8 together with *lox2*, *lox4*, *ubx*, and *abdA* (Supplementary Figs. 5, 6). *Med6* displays high similarity with central Hox genes, sharing all eleven diagnostic residues^31^: Q(6), T(7), R(10), LTR(R/K)RRI(26–32), and E(59) (Supplementary Fig. 8). Notably, *med6* shows posterior-class diagnostic residue (Y at position 20).

*Sce-postC* and *Sce-postD* cluster with the *post1/hox11 – 13* group of the posterior Hox class (Supplementary Figs. 5, 6). Posterior-class residues^31,36^ K(3) and R(18) are present in both, but V(21) is only found in *Sce-postD* (Supplementary Fig. 9). *PostC* also shares as a spiralian *post1* signature^36^ Y(20) with *postA* and *postB* (Supplementary Fig. 9).

### Expression patterns of *Spadella* Hox genes

**Fig. 4:**
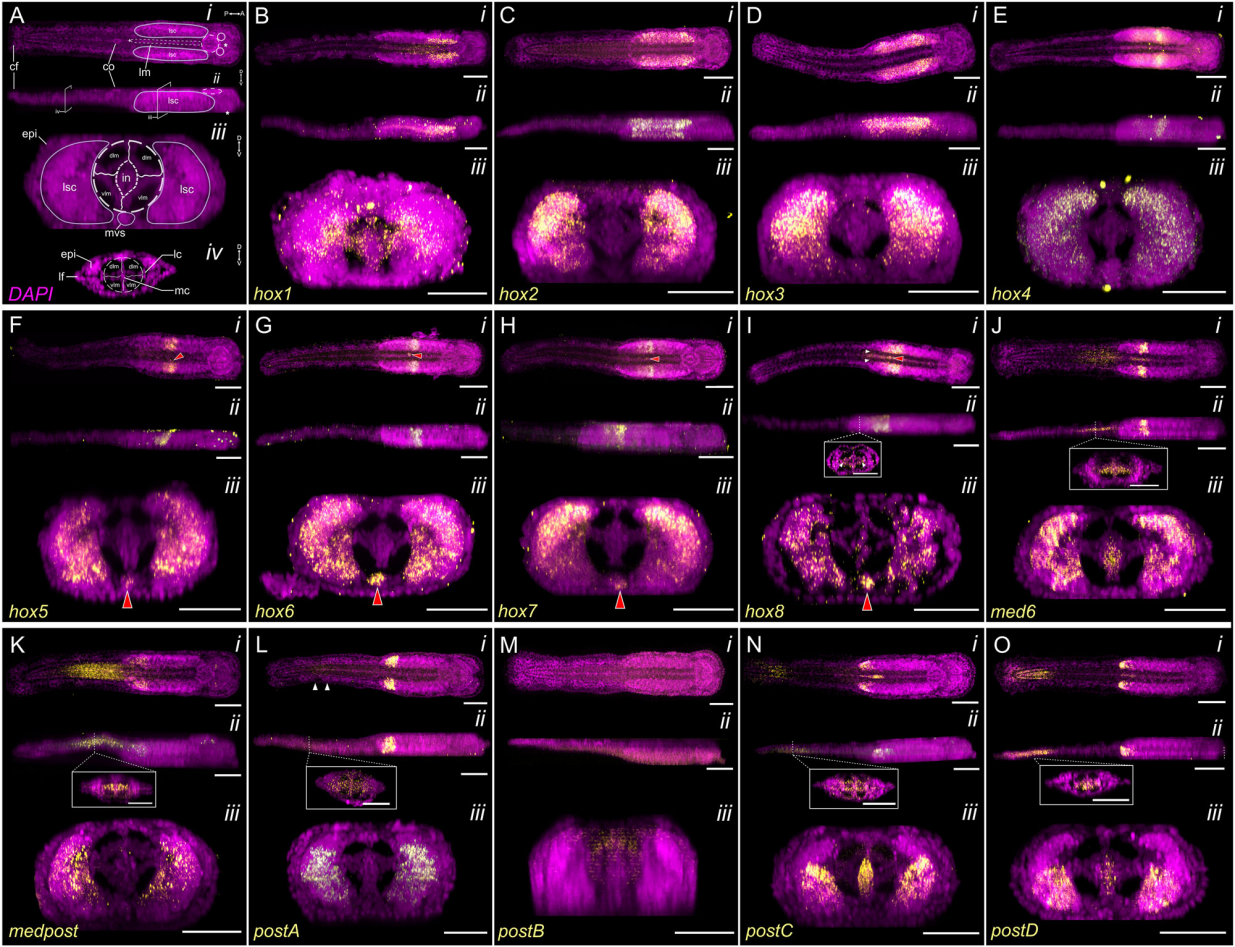
Expression patterns of Hox genes in the hatchling (1 dph) of *Spadella cephaloptera*. Gene transcripts (yellow) are visualized with AlexaFluor-647 (**C**, **J**, **K**, **M**–**O**) or AP-Fast Blue (**B**, **D**–**I**, **L**) and cell nuclei are counterstained with DAPI (purple). **A** General morphology of a hatchling in maximum intensity projection of DAPI-stained whole animal. The eyes are encircled and gut in dashed structure in between the lateral somata clusters (lsc). The position of the mouth and anal opening are indicated by an asterisk and a cross, respectively. (*i*) Dorsal view, (*ii*) lateral view, (*iii*, *iv*) transverse profile of the trunk (*iii*) and the tail (*iv*). **B**–**O** Expression of Hox genes in trunk structures (**B**–**L**, **N**, **O**) and the tail (**J**–**L**, **N**, **O**). **F**–**I** *Sce-hox5* – *Sce-hox8* are expressed in the medioventral somata clusters (*mvs*) (marked with red arrowheads). (**I**i, ii) Expression of *Sce-hox8* in the trunk longitudinal muscle is marked by white arrowheads. (**J**ii – **L**ii, **N**ii, **O**ii) Insets show expression of Hox genes in the epidermis and mesodermal derivatives of the tail. **M** Weak background staining is visible but does not represent specific *Sce-postB* expression. Scale bars: 100 μm, except panels **B**iii–**O**iii (50 μm) and all insets (25 μm). Orientation is indicated in (**A**). cc corona ciliata, cf caudal fin, cg cerebral ganglion, co ciliary tuft/fence organ, dlm dorsal longitudinal muscle, epi epidermis, in intestine, lc lateral cells, lf lateral fin, lm longitudinal muscle, lsc lateral somata clusters, mc mesenterial cells, mvs medioventral somata clusters, vlm ventral longitudinal muscle.

Fluorescent in situ hybridization analyses revealed a staggered spatial and temporal expression pattern of Hox genes along the AP axis during embryonic and post-embryonic development in *S. cephaloptera* (Fig. 1D–G and Fig. 4; Supplementary Fig. 1D–G and Supplementary Fig. 11).

#### Gastrula to early elongation stage

During early-mid gastrulation, *Sce-med6* is expressed in the mid-posterior ectodermal domain (Fig. 1Di, ii; Supplementary Fig. 1Di, ii), expanding by late gastrula to pre-posterior ectoderm, posterior mesoderm, and posterior neuroectoderm (Fig. 1Diii; Supplementary Fig. 1Diii). *Sce-medpost* is expressed in ectodermal and endodermal regions slightly posterior to the embryonic midline in early-mid gastrula (Fig. 1Ei, ii; Supplementary Fig. 1Ei, ii), and by late gastrula in the posterior mesoderm, posterior neuroectoderm, and pre-posterior ectoderm (Fig. 1Eiii; Supplementary Fig. 1Eiii). The posterior Hox genes, *Sce-postC* and *Sce-postD*, are expressed around the blastopore in early-mid gastrula (Fig. 1Fi, Gi, Gii; Supplementary Fig. 1Fi, Gi, Gii), and later expression localizes to the posterior tip of the ectoderm, mesoderm, and neuroectoderm (Fig. 1Fiii, Giii; Supplementary Fig. 1Fiii, Giii). In the early elongation stage, *Sce-med6* and *Sce-medpost* expression occupy mid-posterior trunk and the pre-posterior lateral neuroblasts (Fig. 1Div, Eiv; Supplementary Fig. 1Div, Eiv), whereas *Sce-postC* and *Sce-postD* are restricted to the posterior lateral neuroblasts and the tail bud region (Fig. 1Fiv, Giv; Supplementary Fig. 1Fiv, Giv). *Sce-hox2* expression first appears at early elongation in the anterior and posterior regions of the lateral neuroblast of the nascent VNC (Supplementary Fig. 10).

#### Hatching to juvenile stage

In hatchlings and early juveniles, the lateral somata clusters of the VNC exhibit a staggered Hox patterning (Fig. 4, Supplementary Fig. 11). In the hatchling, *Sce-Hox1* is expressed in the inner anterior regions and in the longitudinal muscles of the trunk (Fig. 4Bi–iii). *Sce-hox2* is broadly expressed except at the anterior and posterior tips (Fig. 4Ci–iii). *Sce-hox3* appears as two vertical bands (Fig. 4Di–iii) overlapping with *Sce-hox2* in the mid-region but extending only slightly before its anterior and posterior boundaries. *Sce-hox4*, *Sce-hox5*, *Sce-hox6*, and *Sce-hox7* expression patterns form horizontal bands in the mid-region with graded overlaps (Fig. 4E–H). *Sce-hox8* is expressed broadly in the posterior lateral somata clusters (Fig. 4Ii–iii), with additional expression in posterior longitudinal muscles around the trunk-tail boundary (white arrowheads in Fig. 4Ii, inset in 4Iii). *Sce-hox5*–*8* are also expressed in the medioventral somata clusters of the VNC (red arrowheads in Fig. 4Fi–Ii, Fiii–Iiii). In the posterior region, *Sce-med6* is expressed as a horizontal band (Fig. 4Ji–iii), while *Sce-medpost* displays a triangular-shaped domain (Fig. 4Ki–iii). Expression of posterior-class genes (*Sce-postA*, *Sce-postC, Sce-postD*) is restricted to the posterior VNC, with the latter two extending slightly further toward the tip (Fig. 4Li–iii, Ni–iii, Oi–iii). Notably, *Sce-postB* is undetectable at all stages examined (Fig. 4Mi–iii). Early juvenile VNC patterns resemble those of hatchlings (Supplementary Fig. 11).

*Sce-hox8*, *Sce-med6*, *Sce-postC, and Sce-postD* are also expressed in the hindgut (Fig. 4Ii, Ji, Ni, Oi). In the tail, a sequential posterior Hox arrangement is observed in hatchlings and early juveniles (Fig. 4Jiii–Liii, Niii, Oiii; Supplementary Fig. 11J, K, M, N). *Sce-med6* is expressed in the anterior tail (Fig. 4Ji, ii; Supplementary Fig. 11J), *Sce-medpost* in the anterior half (Fig. 4Ki, ii; Supplementary Fig. 11K), *Sce-postA* in the pre-posterior end (Fig. 4Li, ii), and both *Sce-postC* and *Sce-postD* in the posterior tail including the caudal fin (Fig. 4Ni, ii, Oi, ii; Supplementary Fig. 11M, N). No expression of *Sce-postA* was detected in the tail of early juveniles (Supplementary Fig. 11L). Hox expression in the tail is mainly observed in longitudinal muscles and faintly visible in epidermal, fin, and mesoderm-derived (e.g. lateral, mesenterial, and medial) cells (inset in Fig. 4Jii – Lii, Nii, Oii).

## Discussion

Our study explores the molecular basis of anterior-posterior axis formation in chaetognaths, a group that is proposed to occupy a Gnathifera-affiliated position within Spiralia^24,27^. Despite their deep evolutionary history, chaetognaths have retained a remarkably conserved, streamlined, tripartite body plan that has undergone minimal morphological divergence over the past 500 million years^68^. Their comparatively conservative body architecture offers a unique opportunity to examine the deployment of axial patterning genes in a lineage with limited body plan divergence. Here, we characterized the expression profiles of AP-patterning genes across multiple, hitherto unexplored, developmental stages.

**Fig. 5:**
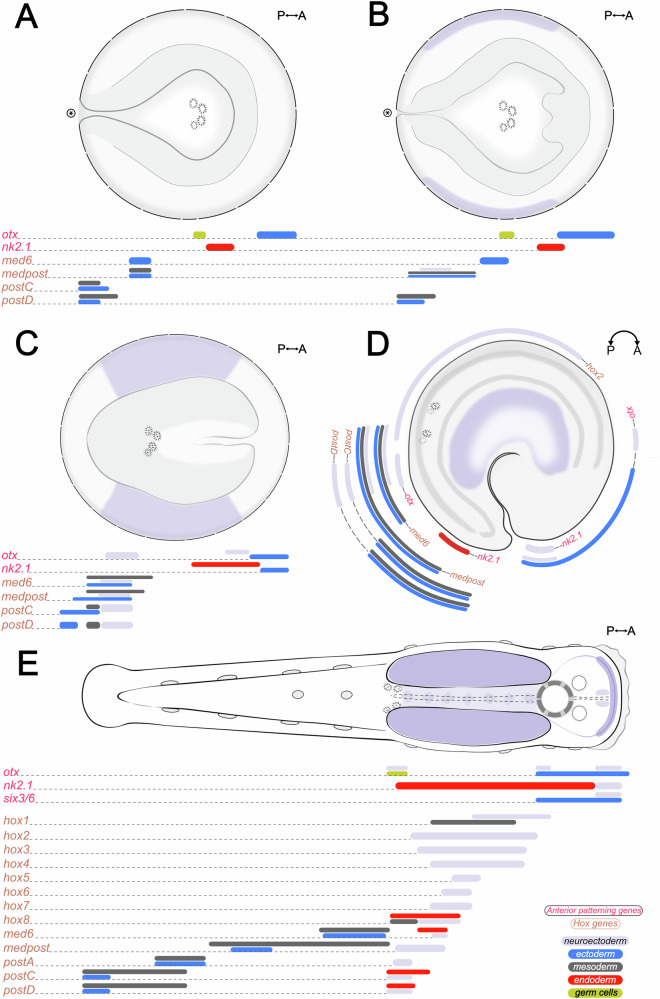
Schematic representation of AP-patterning and Hox gene expression during early development of *Spadella cephaloptera*. **A**–**E** Spatial expression domains of anterior-patterning and Hox genes at key developmental stages: (**A**) early gastrula, (**B**) mid gastrula, (**C**) late gastrula, (**D**) early elongation, and (**E**) hatchling (1 dph). Orientation is indicated in the top-right corner of each panel. Colored bars represent approximate gene expression domains, with bar color denoting germ layer origin: neuroectoderm (light purple), ectoderm (blue), mesoderm (dark gray), endoderm (red), and germ cells (dark yellow).

These data reveal spatially organized domains associated with axial and neural regionalization (Fig. 5), uncovering both ancestral bilaterian features and chaetognath-specific innovations. Our findings provide valuable insights into how chaetognath developmental programs relate to those of other spiralians, contributing to our understanding of conserved and lineage-specific mechanisms of AP patterning in Bilateria.

The expression patterns of the anterior patterning genes *otx*, *six3/6*, and *nk2.1* in *S. cephaloptera* resemble those observed in the anterior-most regions of other bilaterians^13,14,69–73^ (Fig. 5).

*Sce-otx* is consistently expressed in the anterior embryonic domain from gastrula to early elongation. In hatchlings, both *Sce-otx* and *Sce-six3/6* are broadly expressed in anterior regions, consistent with their conserved roles in specifying anterior structures across diverse bilaterian taxa^69,72–79^. Although embryonic data for *Sce-six3/6* are unavailable, its post-embryonic expression remains in the anterior head, in line with its canonical pattern.

In many bilaterians, *six3/6* expression occupies the anterior-most domain with *otx* expression positioned immediately posterior^3,12,73,79–81^. In *Spadella*, however, *Sce-otx* and *Sce-six3/6* overlap at the anterior-most tip of the head, with *Sce-otx* occupying the anterior-most region across stages. This deviation from the common bilaterian arrangement suggests a derived reorganization of *six3/6*-*otx* spatial hierarchy and raises the possibility that the spatial relationship is more evolutionarily labile than previously recognized. Expression of both genes also extends into anterior structures beyond the CNS: *Sce-six3/6* in the eyes, corona ciliata, and anterior epidermis, and *Sce-otx* in cephalic adhesive papillae, oral region, and ciliary sensory organs, reflecting deployment beyond neural territories as observed in many bilaterians^77,79,81–86^. Notably, *Sce-otx* is also expressed in PGCs, a divergence from the canonical bilaterian *otx* patterns that may reflect lineage-specific co-option to germline precursors in *Spadella*.

*Sce-nk2.1* occupies the medial anterior domain of the cerebral ganglion, whereas *Sce-pax6* marks the lateral regions^62^. This mediolateral partitioning resembles patterns known from vertebrates and annelids^87–89^, and is hypothesized as an ancestral bilaterian feature retained in several spiralians, including rotifers^39^ and brachiopods^39,78^, but absent in planarians^90,91^ and the nemertean *Lineus ruber*^39,92^. The mediolateral *nk2.1*–*pax6* subdivision in *S. cephaloptera*, despite their compact hatchling brain, suggests that aspects of ancestral neural patterning can persist in lineages with derived morphologies. Beyond neural structures, *Sce-nk2.1* appears in anterior cells of the endomesoderm of early-mid gastrula, later extending along the developing intestinal rudiment by late gastrula. At hatching, it spans the gut with stronger foregut and hindgut signal, as in annelids^93,94^. By contrast, ecdysozoan and molluscan patterns show foregut-restricted *nk2.1*/*scarecrow* (*scro)*/*ceh-24*^95–97^, whereas chordates express *nk2.1* more broadly across the gut^98–100^. Overall, *nk2.1* gut expression in *S. cephaloptera* is consistent with its roles in gut patterning^39,93,95,96,99,100^ and with its involvement, together with *foxA* and *otx*, in endomesoderm specification across Cnidaria and Bilateria^72,93,101,102^. *Sce-foxA* and *Sce-nk2.1* are expressed in the developing gut^62^ but *Sce-otx* is notably absent, suggesting that *S. cephaloptera* has likely retained only a subset of the core gene regulatory mechanisms associated with endoderm specification and the development of the digestive tract.

Our findings also reveal that the chaetognath *Spadella cephaloptera* possesses a complete set of Hox genes with 14 genes from all paralogous groups, including new members of the central and posterior Hox groups (Fig. 3). By comparison, a recent genomic analysis of the related spadellid *P. gotoi* recovers 15 Hox genes, including additional central- and posterior-class genes, while reportedly lacking *hox3*^42^. However, we detect *hox3* transcripts in publicly available *P. gotoi* RNA-seq data, suggesting that the species likely possesses the full complement. Taken together, these findings represent the largest and most diverse Hox complement reported for protostomes without whole-genome duplication. Among metazoans, *P. gotoi* matches the number of canonical Hox genes (excluding *medpost*) found in the cephalochordate *Branchiostoma* (15 Hox genes)^103^. This stands in contrast to the extensive Hox expansions in many arthropods and vertebrates, which are often associated with genome duplication events^104–106^.

Previous studies identified up to ten chaetognath Hox genes^31,36^. We recovered orthologs of the genes and detected four previously undescribed genes in the group: *Sce-hox2*, *Sce-med6*, *Sce-postC*, and *Sce-postD*. *Sce-hox2* was assigned to PG2 based on orthology inference and diagnostic residues, confirming a complete anterior Hox complement in chaetognaths. No *hox2* and *postD* ortholog was found in other chaetognath transcriptomic datasets potentially due to stage-restricted expression or low sequencing depth (Supplementary Data 1).

The new central-class gene *med6* clusters with *hox8* inside PG8 and retains all the canonical central homeodomain signature. Similar to *hox8*, *med6* also exhibits a divergent UbdA-like parapeptide motif (xxIxELN) typically found in ecdysozoan *ubx*/*abdA* and lophotrochozoan *lox2*/*lox4* orthologs^36^ (Supplementary Fig. 8). Matus et al.^36^ hypothesized that the last common ancestor of protostomes possessed a central-class Hox with the UbdA parapeptide, which subsequently underwent independent duplications and divergence that occurred in Ecdysozoa and Lophotrochozoa, giving rise to *ubx*/*abdA* and *lox2*/*lox4*, respectively. In this framework, after splitting with lophotrochozoans, chaetognath *hox8* and *med6* may also have arisen from a shared ancestral UbdA-bearing central gene followed by lineage-specific modifications that resulted in partial UbdA signal.

We also identified two previously uncharacterized posterior class Hox genes, *Sce-postC* and *Sce-postD*. Both share several plesiomorphic features with ecdysozoan *abdB*, lophotrochozoan *post1* and *post2*, and deuterostome *hox9*–*15* (Supplementary Fig. 9), but lack clear chaetognath-specific motifs. Phylogenetic analyses place them variably: ML recovers *postC/D* as sister to *post1*/*hox11*–*15*, whereas Bayesian inference yields polytomy with *postA* and *postB*, complicating inferences about their evolutionary relationship with other bilaterian posterior Hox genes. The scattered distribution of chaetognath *postA*–*D*, rather than forming a single monophyletic group, suggests lineage-specific duplications of an ancestral posterior gene, followed by sequence divergence that has obscured shared molecular signatures. This pattern resembles Posterior Flexibility, a phenomenon described in deuterostomes where posterior Hox genes show elevated sequence divergence and poor phylogenetic resolution^107^. While Posterior Flexibility has not been formally reported in protostomes, the unresolved affinities of chaetognath *postA*–*D* may reflect similar evolutionary dynamics.

*Sce-postB*, a predicted paralog of *Sce-postA*, was recovered in transcriptomic data and confirmed by PCR, indicating transcriptional activity. However, no expression was detected by FISH at the stages examined, which may reflect low abundance, restricted expression timing, or technical sensitivity limits (e.g. probe inefficiency). Without additional temporal or spatial resolution, the expression dynamics and potential developmental role of *Sce-postB* remain unresolved.

Chaetognaths appear to have retained the ancestral Hox repertoire, supporting the hypothesis of an urbilaterian complement of at least seven Hox genes spanning the anterior, central, and posterior class genes^108,109^. Their Hox complement appears to have expanded through lineage-specific duplication and divergence, yielding one of the most extensive Hox repertoires among bilaterians (Fig. 3). We initially hypothesized that this expansion reflects small-scale events (tandem or segmental duplication) rather than whole-genome duplication (WGD), given the lack of widespread duplication in other transcription factors families (e.g., anterior patterning genes). The *P. gotoi* genome reveals a tandemly duplicated Hox cluster without WGD signatures^42^, reinforcing the view that the expansion of chaetognath Hox likely occurred through local duplication. While *P. gotoi* is not a direct substitute for a *Spadella* genome, its similar Hox content and cluster structure support inferences about expansion mechanisms in the lineage. In contrast, the Rotifera–Acanthocephala clade shows marked reduction with some lineages retaining only one or two anterior-class genes^37^ and despite an ancient WGD in Bdelloidea, they only retain a subset of ancestral Hox genes^110^. These divergent patterns underscore the distinctive chaetognath trajectory in Hox evolution, highlighting their importance for reconstructing the early diversification of bilaterians and contributing to a broader understanding of how Hox gene evolution may have shaped metazoan body plan complexity.

The earliest reported chaetognath Hox expression data came from *SceMed4* (PG5), which was shown to be expressed as two bilateral domains in the VNC of *Spadella*^38^, suggesting a role of Hox genes in patterning the ventral CNS of chaetognaths^38^. Our data extended this view by revealing a staggered expression profile of multiple Hox genes along the AP axis of the VNC in *S. cephaloptera*.

During the late gastrula stage, a subset of central and posterior class Hox genes (*Sce-med6*, *Sce-medpost*, *Sce-postC*, and *Sce-postD*) are broadly expressed in the posterior neuroectoderm (Fig. 5C). By early elongation, these domains resolve into a staggered AP (Fig. 5D), with *Sce-hox2* occupying a more anterior domain in the nascent VNC. This staggered pattern is maintained into hatchlings and juveniles, where 13 Hox genes show distinct expression along the VNC (Fig. 5E). These findings suggest a temporal and spatial continuity of Hox-mediated AP patterning in the developing central nervous system that begins during early neuroectoderm formation and persists through later development.

The AP-staggered pattern reflects a conserved mechanism observed across many bilaterians, in which Hox expression regionalizes the neuroectoderm and developing neural structures during specific developmental stages. In bilaterians, this pattern is evident in the nemertean *Pantinonemertes californiensis* larva^75^, in several molluscan groups^11,111–116^, and annelids^10,117–120^, though often coupled to segmental organization except in the leech *Helobdella* which shows a staggered Hox expression only in ventral neural tissues^121,122^. Hox genes in early development of annelids primarily function in defining overall segment organization and specifying segmental fate rather than being solely involved in patterning the CNS. In the rotifer *Brachionus manjavacas*^16^ and the brachiopod *Terebratalia transversa*^19^, only a partial resemblance of staggered patterning can be observed^11^. Outside Spiralia, staggered expression regionalizes, to varying degrees, the developing CNS in arthropods^8,123,124^, a hemichordate^5^, chordates^9,125,126^, and acoels^127,128^.

In *S. cephaloptera*, the staggered Hox expression in the VNC may also contribute to regional specialization^38^, potentially influencing the differentiation of distinct neuronal subtypes along the AP axis, similar to how Hox genes regulate region-specific neuronal fates in *Drosophila*^8,129^, *C. elegans*^130^, and vertebrates^131^. Although direct evidence for a spatial correlation between neuronal diversity and Hox gene expression domains in chaetognaths is currently unavailable, the arrangement of RFamide-immunoreactive (ir) neurons along the AP axis of the VNC offers a potential framework for speculation. RFamine-ir neurons are serially arranged, with lateral cells (L-group) located anteriorly and dorsal cells (D-group) positioned centrally (D1 – D5) and posteriorly (D6, D7, and the giant X neuron found only in the Spadellidae)^22,132–135^. Conceptually mapping these onto Hox domains, it is plausible that L-group neurons nest within *Sce-hox1-3* domains, midline G-groups with central-class domains, and posterior neurons with *Sce-medpost* and posterior-class domains. If such spatial alignment reflects underlying functional relationships, it would support the hypothesis that Hox genes contribute to neural subtype patterning in chaetognaths, as in other bilaterians. This underscores the need for future studies on chaetognath neuronal diversity and the developmental mechanisms guiding its specification.

*P. gotoi* exhibits an AP-staggered patterning for a subset of central- and posterior-class Hox genes in the VNC of early juveniles^42^, paralleling our findings in *Spadella*^41^. However, the reported expression patterns of some genes (e.g., *Pgo-hox1* and *Pgo-medpost*) appear broader (VNC) or differ in tissue association (e.g., ciliary fence receptors), which may reflect species-specific differences, developmental staging, or technical variation (e.g., chromogenic ISH versus HCR-FISH).

The spatially staggered expression of Hox genes in the VNC of *S. cephaloptera* aligns chaetognaths with a diverse set of bilaterians exhibiting similar AP neural patterning, including annelids, molluscs, arthropods, and vertebrates. Given that chaetognaths represent one of the earliest-diverging lineages within Spiralia, these findings considerably broaden the phylogenetic scope of Hox-mediated nervous system regionalization. This supports the long-standing view that staggered Hox expression along the AP axis is an ancestral bilaterian trait, likely present in early spiralians. While Hox-mediated neural patterning is broadly conserved, the extent and precision of staggered patterns vary across lineages and developmental stages (e.g., partial or transient patterns in rotifers and brachiopods^16,69^ and departures from expected axial position in certain molluscs^116^). This variation may reflect lineage-specific modifications or reductions of an ancestral patterning mechanism, underscoring the significance of chaetognaths in preserving a complete, temporally sustained, and spatially ordered deployment of Hox genes during nervous system development. This conservation, along with the conserved bauplan since the early Cambrian, raises the possibility that chaetognaths retain a neural Hox expression pattern more reflective of the ancestral spiralian, or even bilaterian, condition. Conversely, if chaetognaths independently recruited Hox genes for nervous system patterning, it would represent a striking case of parallel evolution involving a deeply conserved genetic toolkit. Consequently, these findings position chaetognaths as a critical group for reconstructing early bilaterian nervous system evolution.

Hox genes, however, are not restricted to neural tissues and are also expressed in non-neural domains. From gastrula stages onward, *Sce-med6*, *Sce-medpost*, *Sce-postC*, and *Sce-postD* are expressed in the posterior mesoderm and ectoderm in spatially staggered domains largely confined to the posterior half of the embryo (Fig. 5A–D). In hatchlings, several Hox genes are expressed in non-neural structures of the trunk, including longitudinal musculature and the posterior region of the endoderm-derived intestine (Fig. 4), suggesting potential roles in mesodermal and endodermal regionalization.

In the trunk musculature, *Sce-hox1* marks the mid-section longitudinal muscles (weakening posteriorly), while *Sce-hox8* is confined to posterior longitudinal muscles. The absence of other Hox gene expression domains in the trunk musculature suggests a limited Hox involvement in mesodermal patterning at this stage. Since *hox1*/*labial* and *hox8/lox2/lox4* orthologs are not typically implicated in mesodermal patterning in other spiralians, their expression here may reflect co-option for *S. cephaloptera* trunk muscle development. Although, it remains unclear whether this represents chaetognath-specific adaptation or an underexplored spiralian role.

Several Hox genes *(Sce-hox8*, *Sce-med6*, *Sce-postC*, and *Sce-postD*) are also expressed in the posterior intestine of the hatchling, overlapping with previously described zones of cell-type specialization: anterior light-granule cells (luminal digestion) and posterior dark-granule cells (absorption and intracellular digestion)^136,137^. The posterior localization of *Sce-postC* and *Sce-postD*, particularly at the rectal terminus, also suggests roles in hindgut regionalization and anus formation. Posterior Hox genes expression in the hindgut also occurs in *Drosophila*^138^, the tunicate *Ciona intestinalis*^125^, the sea cucumber *Apostichopus japonicus*^139^, and vertebrates (e.g.^140,141^). Hox genes such as *hox3, hox6*/*lox5*, *hox7*/*antp* are expressed in specific gut domains in the scaphopod *Antalis entalis*^116^ and in the annelid *Capitella*^10^. Taken together, these findings suggest that Hox genes may contribute to hindgut regionalization in *S. cephaloptera*, and that Hox recruitment to the gut may have repeatedly occurred across bilaterians. Whether this reflects shared ancestry or convergent co-option remains unresolved.

Such tissue-specific roles naturally prompt questions about the underlying genomic organization of chaetognath Hox genes. Colinearity in *S. cephaloptera* remains untested because the genomic Hox gene order is unknown. The genome of *P. gotoi* reveals a physically linked Hox cluster and support a spatial colinearity in early juvenile VNC^42^. This raises the possibility that a similar genomic organization underlies the VNC-wide AP-staggered deployment across most Hox loci observed in *S. cephaloptera*. Our embryonic expression data, however, point to a possible disruption of temporal colinearity. *Sce-med6*, *Sce-medpost*, *Sce-postC*, and *Sce-postD* are detected at the gastrula stage, while the anterior-class Hox gene *Sce-hox2* only initiates during early elongation. Because this inference is based on a single anterior gene, temporal data for the full Hox set are needed to evaluate the broader pattern. Overall, these findings suggest that chaetognaths retain spatial aspects of ancestral Hox regulation while exhibiting modifications in their temporal deployment, consistent with partial decoupling of spatial and temporal colinearity observed in some bilaterians^5,119,142,143^.

Although *S. cephaloptera* has been suggested to belong to a morphologically derived benthic lineage (Spadellidae)^144^, its complete Hox complement and AP-staggered deployment offer a useful comparative baseline for inferring posterior patterning in Chaetognatha. Central and posterior Hox genes (*Sce-med6*, *Sce-medpost*, *Sce-postA*, *Sce-postC*, and *Sce-postD*) exhibit spatially staggered expression along the postanal tail (Fig. 5E), consistent with their involvement in caudal regionalization. During embryogenesis, these genes (except *Sce-postA*) are expressed in the posterior epidermal and mesodermal cells from gastrulation to early elongation stages, consistent with their later localization in the hatchling tail. Their dual localization in the posterior VNC and tail is more consistent with pleiotropy and tissue-specific readouts of a single AP axis rather than axis duplication, as no ectopic anterior markers (e.g., *six3/6*, *otx*, *nk2.1*) or locally replicated Hox series (full complement) are detected outside the VNC. Moreover, the restriction of extra non-neural domains to posterior-side genes is consistent with Posterior Flexibility, wherein posterior-class Hox genes are coopted into lineage-specific morphological elaborations, particularly in appendages and posterior structures^145^. However, whether these deployments are mechanistically decoupled remains to be investigated.

Postanal tails are rare among bilaterians and have only been described in chordates and chaetognaths. In chordates, postanal tail development involves posterior-class Hox genes (*hox9–13*), together with regulatory factors such as *sonic hedgehog* (*shh*) and *brachyury* (*bra/tbxt*)^146–148^. While this raises the possibility of functional parallels, no *shh* ortholog is detected in *S. cephaloptera* transcriptome, and *bra* expression in *P. gotoi* is restricted to anterior embryonic regions, particularly the stomodeum and presumptive mouth^149^. Combined with deep phylogenetic divergence and presumed differences in cell lineage, these observations favor independent origin of chaetognath and chordate postanal tails rather than a shared ancestral program.

Further support comes from comparisons with monogonont rotifers, the closest gnathiferan relatives for which Hox gene expression data are available. Across the Rotifera–Acanthocephala clade, posterior Hox genes are absent, and in monogononts, Hox expression is restricted to neural tissues and their postanal extension (i.e. foot) is not patterned by Hox genes^16,37,110^. Consequently, these comparisons suggest that postanal tails of chaetognaths arose via independent co-option of posterior Hox genes to pattern novel caudal structures.

Fossil data provide crucial context for understanding the evolutionary origins of the postanal tail in chaetognaths. Stem-group chaetognaths (e.g. *Amiskwia sagittiformis* and *Timorbestia koprii*), exhibit a subterminal anus with a short postanal extension (caudal fin)^150,151^, while early crown-group taxa from the early Cambrian (e.g. *Protosagitta spinosa* and *Capinatator praetermissus*) possess a more elongate tail posterior to the anus^152,153^, suggesting tail elongation emerged within the crown chaetognath lineage. Variation among modern taxa suggests ecological or functional pressures may have shaped tail morphology. The staggered deployment of posterior Hox genes in the tail, together with its persistence in both fossils and extant species, supports the hypothesis that this structure emerged through lineage-specific co-option of ancestral Hox-based patterning, contributing to a novel posterior anatomy.

Despite its derived ecology, *S. cephaloptera* retains a postanal tail and staggered posterior Hox expression, providing a comparative system for investigating Hox roles in posterior elaboration. The correspondence between morphological persistence and posterior-Hox deployment is compatible with the hypothesis of conserved developmental program, but comparative expression and functional tests are required to assess whether a shared mechanism underlies tail maintenance across Chaetognatha.

## Conclusion

Our study provides a comprehensive analysis of anterior-patterning (*otx, nk2.1, six3/6*) and Hox gene expression in the chaetognath *Spadella cephaloptera*, providing new insights into the AP axis patterning in this lineage. We show that anterior patterning genes are expressed in the cerebral ganglion and associated head structures, supporting their conserved role in anterior CNS development across Bilateria. In addition, we document a staggered expression of Hox genes, including the newly described *Sce-med6*, *Sce-postC*, and *Sce-postD*, along the VNC, hindgut, and postanal tail. These patterns suggest that chaetognaths maintain core bilaterian AP patterning programs while also deploying lineage-specific modifications. The VNC regionalization parallels neural patterning observed in other bilaterians, highlighting shared developmental mechanisms despite divergent morphologies. As a lineage occupying a key phylogenetic position within Spiralia, chaetognaths provide a critical comparative perspective for reconstructing spiralian evolution. Their retention of a complete Hox cluster, in contrast to the reduced Rotifera-Acanthocephala repertoire, underscores the diversity of Hox gene utilization across Spiralia. By addressing a long-standing gap in spiralian evo-devo, this study establishes chaetognaths as an essential model for reconstructing ancestral spiralian developmental programs and for understanding how conserved genetic toolkits are independently co-opted and elaborated across animal evolution.

## Supplementary information

Supplementary Information

Description of Additional Supplementary Files

Supplementary Data 1

Reporting Summary

## Data Availability

The assembled *Spadella cephaloptera* transcriptome is available on Zenodo under the accession 10.5281/zenodo.7602960^44^. Newly obtained *S. cephaloptera* sequence data that support the findings of this study have been deposited to GenBank with accession numbers: PX219847-PX219863. All other data supporting the findings of this study are available from the corresponding authors upon reasonable request.

## Code availability

All scripts used to download, assemble, and annotate transcriptomes for Hox gene mining are available at GitHub (https://github.com/jfordonez/ChaetognathAPPatterning.git). The exact version of the code used in this study has been archived on Zenodo under the 10.5281/zenodo.17705168^154^.
