# Supplementary material for: Anterior-posterior patterning in the chaetognath Spadella cephaloptera informs bilaterian nervous system and tail evolution
Source: Commun Biol. 2025 Dec 26;9:122. doi: 10.1038/s42003-025-09398-6 (PMC12852818; doi:10.1038/s42003-025-09398-6)
Supplement: Supplementary file 1 — Supplementary Information [file 42003_2025_9398_MOESM1_ESM.pdf]

Supplementary Information

Anterior-posterior patterning in the chaetognath *Spadella cephaloptera* informs bilaterian nervous system and tail evolution

June F. Ordoñez[1,2,*], Tim Wollesen[1,*]

1 Unit for Integrative Zoology, Department of Evolutionary Biology, University of Vienna, 1030 Vienna, Austria

2 Vienna Doctoral School of Ecology and Evolution (VDSEE), University of Vienna, 1030 Vienna, Austria

*Corresponding authors

Tim Wollesen

Email address: tim.wollesen@univie.ac.at

June F. Ordoñez

Email address: june.ordonez@univie.ac.at

Supplementary tables

Supplementary Table 1. GenBank accession numbers of all sequences used in this study. Species used as outgroup for each gene is highlighted in yellow. The substitution model used in the phylogenetic analysis is also indicated.

**Hox**	**Substitution model: VT + G + F**				
**Accession**	**Gene ID**	***Species***	**Sample ID**	**Phylum**	**SubGroup (Subphylum/Class/Clade)**
APD15641.1	Hox1	*Acanthochitona crinita*	Acr_Hox1	Mollusca	Polyplacophora
APD15642.1	Hox2	*Acanthochitona crinita*	Acr_Hox2	Mollusca	Polyplacophora
APD15643.1	Hox3	*Acanthochitona crinita*	Acr_Hox3	Mollusca	Polyplacophora
APD15644.1	Hox4	*Acanthochitona crinita*	Acr_Hox4	Mollusca	Polyplacophora
APD15645.1	Hox5	*Acanthochitona crinita*	Acr_Hox5	Mollusca	Polyplacophora
APC93964.1	Hox7	*Acanthochitona crinita*	Acr_Hox7	Mollusca	Polyplacophora
APD15647.1	Lox2	*Acanthochitona crinita*	Acr_Lox2	Mollusca	Polyplacophora
APD15648.1	Lox4	*Acanthochitona crinita*	Acr_Lox4	Mollusca	Polyplacophora
APD15646.1	Lox5	*Acanthochitona crinita*	Acr_Lox5	Mollusca	Polyplacophora
APD15649.1	Post2	*Acanthochitona crinita*	Acr_Post2	Mollusca	Polyplacophora
AAF81909.1	Hox11	*Branchiostoma floridae*	Bfl_Hox11	Chordata	Cephalochordata
ACJ74382.1	Hox1	*Branchiostoma lanceolatum*	Bla_Hox1	Chordata	Cephalochordata
ACJ74389.1	Hox10	*Branchiostoma lanceolatum*	Bla_Hox10	Chordata	Cephalochordata
ACJ74391.1	Hox12	*Branchiostoma lanceolatum*	Bla_Hox12	Chordata	Cephalochordata
ACJ74390.1	Hox13	*Branchiostoma lanceolatum*	Bla_Hox13	Chordata	Cephalochordata
ACJ74393.1	Hox14	*Branchiostoma lanceolatum*	Bla_Hox14	Chordata	Cephalochordata
ACJ74394.1	Hox15	*Branchiostoma lanceolatum*	Bla_Hox15	Chordata	Cephalochordata
ACJ74381.1	Hox2	*Branchiostoma lanceolatum*	Bla_Hox2	Chordata	Cephalochordata
ACJ74380.1	Hox3	*Branchiostoma lanceolatum*	Bla_Hox3	Chordata	Cephalochordata
ACJ74383.1	Hox4	*Branchiostoma lanceolatum*	Bla_Hox4	Chordata	Cephalochordata
ACJ74385.1	Hox5	*Branchiostoma lanceolatum*	Bla_Hox5	Chordata	Cephalochordata
ACJ74384.1	Hox6	*Branchiostoma lanceolatum*	Bla_Hox6	Chordata	Cephalochordata
ACJ74388.1	Hox7	*Branchiostoma lanceolatum*	Bla_Hox7	Chordata	Cephalochordata
ACJ74387.1	Hox8	*Branchiostoma lanceolatum*	Bla_Hox8	Chordata	Cephalochordata
ACJ74386.1	Hox9	*Branchiostoma lanceolatum*	Bla_Hox9	Chordata	Cephalochordata
APD78496.1	Hox2	*Brachionus manjavacas*	Bma_Hox2	Gnathifera	Rotifera
APD78497.1	Hox3	*Brachionus manjavacas*	Bma_Hox3	Gnathifera	Rotifera
APD78498.1	Hox4	*Brachionus manjavacas*	Bma_Hox4	Gnathifera	Rotifera
RNA18956.1	Hox5	*Brachionus manjavacas*	Bma_Hox5	Gnathifera	Rotifera
APD78499.1	Hox6	*Brachionus manjavacas*	Bma_Hox6	Gnathifera	Rotifera
APD78500.1	MedPost	*Brachionus manjavacas*	Bma_MedPost	Gnathifera	Rotifera
CAA07502.1	AbdA	*Cupiennius salei*	Csa_AbdA	Arthropoda	Chelicerata
CAB40807.1	AbdB	*Cupiennius salei*	Csa_AbdB	Arthropoda	Chelicerata

CAA07499.1	Antp	*Cupiennius salei*	Csa_Antp	Arthropoda	Chelicerata
CAA07498.1	Dfd	*Cupiennius salei*	Csa_Dfd	Arthropoda	Chelicerata
CAA06645.1	Hox3	*Cupiennius salei*	Csa_Hox3	Arthropoda	Chelicerata
CAA07497.1	Lab	*Cupiennius salei*	Csa_Lab	Arthropoda	Chelicerata
CAL91855.1	Pb	*Cupiennius salei*	Csa_Pb	Arthropoda	Chelicerata
CAL91856.1	Scr	*Cupiennius salei*	Csa_Scr	Arthropoda	Chelicerata
CAA07500.1	Ubx1	*Cupiennius salei*	Csa_Ubx1	Arthropoda	Chelicerata
ABY67962.1	Antp	*Capitella teleta*	Cte_Antp	Annelida	Polychaeta
ABY67955.1	Dfd	*Capitella teleta*	Cte_Dfd	Annelida	Polychaeta
ABG82164.1	Evx	*Capitella teleta*	Cte_Evx	Annelida	Polychaeta
ABY67954.1	Hox3	*Capitella teleta*	Cte_Hox3	Annelida	Polychaeta
ABY67952.1	Lab	*Capitella teleta*	Cte_Lab	Annelida	Polychaeta
ABY67959.1	Lox2	*Capitella teleta*	Cte_Lox2	Annelida	Polychaeta
ABY67958.1	Lox4	*Capitella teleta*	Cte_Lox4	Annelida	Polychaeta
ABY67957.1	Lox5	*Capitella teleta*	Cte_Lox5	Annelida	Polychaeta
ABY67953.1	Pb	*Capitella teleta*	Cte_Pb	Annelida	Polychaeta
ABY67961.1	Post1	*Capitella teleta*	Cte_Post1	Annelida	Polychaeta
ABY67960.1	Post2	*Capitella teleta*	Cte_Post2	Annelida	Polychaeta
ABY67956.1	Scr	*Capitella teleta*	Cte_Scr	Annelida	Polychaeta
BAA77405.1	PLox5	*Dugesia japonica*	Dja_PLox5	Platyhelminthes	
BAA77406.1	PLox6	*Dugesia japonica*	Dja_PLox6	Platyhelminthes	
CCK73377.1	AbdA	*Euperipatoides kanangrensis*	Eka_AbdA	Onychophora	
CCK73379	AbdB	*Euperipatoides kanangrensis*	Eka_AbdB	Onychophora	
CCK73375	Antp	*Euperipatoides kanangrensis*	Eka_Antp	Onychophora	
CCK73372	Dfd	*Euperipatoides kanangrensis*	Eka_Dfd	Onychophora	
CCK73374.1	Ftz	*Euperipatoides kanangrensis*	Eka_Ftz	Onychophora	
CCK73371	Hox3	*Euperipatoides kanangrensis*	Eka_Hox3	Onychophora	
CCK73369.1	Lab	*Euperipatoides kanangrensis*	Eka_Lab	Onychophora	
CCK73370.1	Pb	*Euperipatoides kanangrensis*	Eka_Pb	Onychophora	
CCK73373	Scr	*Euperipatoides kanangrensis*	Eka_Scr	Onychophora	
CCK73376	Ubx1	*Euperipatoides kanangrensis*	Eka_Ubx1	Onychophora	
AAL25809	Antp	*Euprymna scolopes*	Esc_Antp	Mollusca	Cephalopoda
AAL25806.1	Hox3	*Euprymna scolopes*	Esc_Hox3	Mollusca	Cephalopoda
AAL25804.1	Lab	*Euprymna scolopes*	Esc_Lab	Mollusca	Cephalopoda
AAL25810.1	Lox4	*Euprymna scolopes*	Esc_Lox4	Mollusca	Cephalopoda
AAL25808.1	Lox5	*Euprymna scolopes*	Esc_Lox5	Mollusca	Cephalopoda
AAL25811.1	Post1	*Euprymna scolopes*	Esc_Post1	Mollusca	Cephalopoda
AAL25812	Post2	*Euprymna scolopes*	Esc_Post2	Mollusca	Cephalopoda
AAL25807.1	Scr	*Euprymna scolopes*	Esc_Scr	Mollusca	Cephalopoda
ABS18809	Hox1	*Flaccisagitta enflata*	Fen_Hox1	Chaetognatha	
ABS18810.1	Hox3	*Flaccisagitta enflata*	Fen_Hox3	Chaetognatha	
ABS18811.1	Hox4	*Flaccisagitta enflata*	Fen_Hox4	Chaetognatha	
ABS18812	Hox6	*Flaccisagitta enflata*	Fen_Hox6	Chaetognatha	
ABS18813	Hox7	*Flaccisagitta enflata*	Fen_Hox7	Chaetognatha	
ABS18814	Hox8	*Flaccisagitta enflata*	Fen_Hox8	Chaetognatha	

ABS18817	MedPost	*Flaccisagitta enflata*	Fen_MedPost	Chaetognatha	
ABS18815	PostA	*Flaccisagitta enflata*	Fen_PostA	Chaetognatha	
ABS18816	PostB	*Flaccisagitta enflata*	Fen_PostB	Chaetognatha	
QFQ66879.1	Hox1	*Hofstenia miamia*	Hmi_Hox1	Xenacoelomorpha	
CAA78665	Lox2	*Helobdella robusta*	Hro_Lox2	Annelida	Clitellata
NP_001291448.1	Evx-1	*Homo sapiens*	Hsa_Evx-1	Chordata	Vertebrata
NP_001073927.1	Evx-2	*Homo sapiens*	Hsa_Evx-2	Chordata	Vertebrata
AAB35423.2	*HoxA1*	*Homo sapiens*	Hsa_HoxA1	Chordata	Vertebrata
NP_005514.1	*HoxA11*	*Homo sapiens*	Hsa_HoxA11	Chordata	Vertebrata
AAC50993.1	*HoxA13*	*Homo sapiens*	Hsa_HoxA13	Chordata	Vertebrata
NP_006726.1	*HoxA2*	*Homo sapiens*	Hsa_HoxA2	Chordata	Vertebrata
NP_705895.1	*HoxA3*	*Homo sapiens*	Hsa_HoxA3	Chordata	Vertebrata
NP_002132.3	*HoxA4*	*Homo sapiens*	Hsa_HoxA4	Chordata	Vertebrata
CAG47052.1	*HoxA5*	*Homo sapiens*	Hsa_HoxA5	Chordata	Vertebrata
NP_076919.1	*HoxA6*	*Homo sapiens*	Hsa_HoxA6	Chordata	Vertebrata
CAA06713.1	*HoxA7*	*Homo sapiens*	Hsa_HoxA7	Chordata	Vertebrata
NP_689952.1	*HoxA9*	*Homo sapiens*	Hsa_HoxA9	Chordata	Vertebrata
AAH70233.1	*HoxB13*	*Homo sapiens*	Hsa_HoxB13	Chordata	Vertebrata
NP_002136.1	*HoxB2*	*Homo sapiens*	Hsa_HoxB2	Chordata	Vertebrata
AAD10852.1	*HoxB3*	*Homo sapiens*	Hsa_HoxB3	Chordata	Vertebrata
AAG45052.1	*HoxB4*	*Homo sapiens*	Hsa_HoxB4	Chordata	Vertebrata
NP_002138.1	*HoxB5*	*Homo sapiens*	Hsa_HoxB5	Chordata	Vertebrata
NP_061825.2	*HoxB6*	*Homo sapiens*	Hsa_HoxB6	Chordata	Vertebrata
NP_004493.3	*HoxB7*	*Homo sapiens*	Hsa_HoxB7	Chordata	Vertebrata
AAG42143.1	*HoxB8*	*Homo sapiens*	Hsa_HoxB8	Chordata	Vertebrata
AAG42144.1	*HoxB9*	*Homo sapiens*	Hsa_HoxB9	Chordata	Vertebrata
NP_059105.2	*HoxC10*	*Homo sapiens*	Hsa_HoxC10	Chordata	Vertebrata
NP_055027.1	*HoxC11*	*Homo sapiens*	Hsa_HoxC11	Chordata	Vertebrata
AAK16717.1	*HoxC12*	*Homo sapiens*	Hsa_HoxC12	Chordata	Vertebrata
AAF73439.1	*HoxC13*	*Homo sapiens*	Hsa_HoxC13	Chordata	Vertebrata
AAG42145.1	*HoxC4*	*Homo sapiens*	Hsa_HoxC4	Chordata	Vertebrata
EAW96748.1	*HoxC5*	*Homo sapiens*	Hsa_HoxC5	Chordata	Vertebrata
CAG33235.1	*HoxC6*	*Homo sapiens*	Hsa_HoxC6	Chordata	Vertebrata
AAG42146.1	*HoxC8*	*Homo sapiens*	Hsa_HoxC8	Chordata	Vertebrata
AAG42151.1	*HoxC9*	*Homo sapiens*	Hsa_HoxC9	Chordata	Vertebrata
AAG44444.1	*HoxD1*	*Homo sapiens*	Hsa_HoxD1	Chordata	Vertebrata
NP_002139.2	*HoxD10*	*Homo sapiens*	Hsa_HoxD10	Chordata	Vertebrata
AAF79045.1	*HoxD11*	*Homo sapiens*	Hsa_HoxD11	Chordata	Vertebrata
AAF79044.1	*HoxD12*	*Homo sapiens*	Hsa_HoxD12	Chordata	Vertebrata
AAC51635.1	*HoxD13*	*Homo sapiens*	Hsa_HoxD13	Chordata	Vertebrata
CAA71102.1	*HoxD3*	*Homo sapiens*	Hsa_HoxD3	Chordata	Vertebrata
NP_055436.2	*HoxD4*	*Homo sapiens*	Hsa_HoxD4	Chordata	Vertebrata
AAG42152.1	*HoxD8*	*Homo sapiens*	Hsa_HoxD8	Chordata	Vertebrata
NP_055028.3	*HoxD9*	*Homo sapiens*	Hsa_HoxD9	Chordata	Vertebrata
ACM69148.1	Hox5	*Isodiametra pulchra*	Ipu_Hox5	Xenacoelomorpha	
AAD45590	Antp	*Lingula anatina*	Lan_Antp	Brachiopoda	Lingulata
AAD45588	Hox3	*Lingula anatina*	Lan_Hox3	Brachiopoda	Lingulata
AAD45587	Lab	*Lingula anatina*	Lan_Lab	Brachiopoda	Lingulata
AAD45592	Lox2	*Lingula anatina*	Lan_Lox2	Brachiopoda	Lingulata

AAD45593	Lox4	*Lingula anatina*	Lan_Lox4	Brachiopoda	Lingulata
AAD45591	Lox5	*Lingula anatina*	Lan_Lox5	Brachiopoda	Lingulata
AAD45594	Post1	*Lingula anatina*	Lan_Post1	Brachiopoda	Lingulata
AAD45595	Post2	*Lingula anatina*	Lan_Post2	Brachiopoda	Lingulata
AAD45589	Scr	*Lingula anatina*	Lan_Scr	Brachiopoda	Lingulata
ALQ28240.1	Hox3	*Loxosomella murmanica*	Lmu_Hox3	Entoprocta	Loxosomatidae
ALQ28241.1	Lab	*Loxosomella murmanica*	Lmu_Lab	Entoprocta	Loxosomatidae
ALQ28242.1	Lox4	*Loxosomella murmanica*	Lmu_Lox4	Entoprocta	Loxosomatidae
ALQ28243.1	Lox5	*Loxosomella murmanica*	Lmu_Lox5	Entoprocta	Loxosomatidae
ALQ28244.1	Post2	*Loxosomella murmanica*	Lmu_Post2	Entoprocta	Loxosomatidae
PMC5347542	Lox4	*Lepidodermella squamata*	Lsq_Lox4	Gastrotricha	
AKE07577	Antp	*Maculaura alaskensis*	Mal_Antp	Nemertea	Pilidiophora
AKE07586	Dfd	*Maculaura alaskensis*	Mal_Dfd	Nemertea	Pilidiophora
AKE07579	Hox3	*Maculaura alaskensis*	Mal_Hox3	Nemertea	Pilidiophora
AKE07580	Lab	*Maculaura alaskensis*	Mal_Lab	Nemertea	Pilidiophora
AKE07581	Lox4	*Maculaura alaskensis*	Mal_Lox4	Nemertea	Pilidiophora
AKE07585	Lox5	*Maculaura alaskensis*	Mal_Lox5	Nemertea	Pilidiophora
AKE07582	Pb	*Maculaura alaskensis*	Mal_Pb	Nemertea	Pilidiophora
AKE07584	Post2	*Maculaura alaskensis*	Mal_Post2	Nemertea	Pilidiophora
AKE07583	Scr	*Maculaura alaskensis*	Mal_Scr	Nemertea	Pilidiophora
AAD40649	AdbB	*Priapulus caudatus*	Pca_AdbB	Priapulida	Priapulimorpha
AAD40643	Dfd	*Priapulus caudatus*	Pca_Dfd	Priapulida	Priapulimorpha
AAD40644	HB1	*Priapulus caudatus*	Pca_HB1	Priapulida	Priapulimorpha
AAD40645	HB2	*Priapulus caudatus*	Pca_HB2	Priapulida	Priapulimorpha
AAD40646	HB3	*Priapulus caudatus*	Pca_HB3	Priapulida	Priapulimorpha
AAD40648	HB4	*Priapulus caudatus*	Pca_HB4	Priapulida	Priapulimorpha
AAD40650	HB5	*Priapulus caudatus*	Pca_HB5	Priapulida	Priapulimorpha
AAD40642	Hox3	*Priapulus caudatus*	Pca_Hox3	Priapulida	Priapulimorpha
AAD40640	Lab	*Priapulus caudatus*	Pca_Lab	Priapulida	Priapulimorpha
AAD40641	Pb	*Priapulus caudatus*	Pca_Pb	Priapulida	Priapulimorpha
AAD40647	Ubx1	*Priapulus caudatus*	Pca_Ubx1	Priapulida	Priapulimorpha
QID57590.1	Antp	*Phoronopsis harmeri*	Pha_Antp	Phoronida	Phoronidae
QID57588.1	Dfd	*Phoronopsis harmeri*	Pha_Dfd	Phoronida	Phoronidae
QID57587.1	Hox3	*Phoronopsis harmeri*	Pha_Hox3	Phoronida	Phoronidae
QID57585.1	Lab	*Phoronopsis harmeri*	Pha_Lab	Phoronida	Phoronidae
QID57591.1	Lox4	*Phoronopsis harmeri*	Pha_Lox4	Phoronida	Phoronidae
QID57589.1	Lox5	*Phoronopsis harmeri*	Pha_Lox5	Phoronida	Phoronidae
QID57586.1	Pb	*Phoronopsis harmeri*	Pha_Pb	Phoronida	Phoronidae
QID57592.1	Post2	*Phoronopsis harmeri*	Pha_Post2	Phoronida	Phoronidae
Saadi et al. 2023[1]	DfdaA	*Stephanella hina*	Shi_DfdaA	Bryozoa	Phylactolaemata
Saadi et al. 2023[1]	DfdaB	*Stephanella hina*	Shi_DfdaB	Bryozoa	Phylactolaemata
Saadi et al. 2023[1]	Lox4	*Stephanella hina*	Shi_Lox4	Bryozoa	Phylactolaemata
Saadi et al. 2023[1]	Lox5a	*Stephanella hina*	Shi_Lox5a	Bryozoa	Phylactolaemata
Saadi et al. 2023[1]	Lox5b	*Stephanella hina*	Shi_Lox5b	Bryozoa	Phylactolaemata
Saadi et al. 2023[1]	Pb	*Stephanella hina*	Shi_Pb	Bryozoa	Phylactolaemata
Saadi et al. 2023[1]	Post2	*Stephanella hina*	Shi_Post2	Bryozoa	Phylactolaemata
NP_001164694.1	Evx	*Saccoglossus kowalevskii*	Sko_Evx	Hemichordata	Enteropneusta
AAP79296	Hox1	*Saccoglossus kowalevskii*	Sko_Hox1	Hemichordata	Enteropneusta
ABK00022	Hox11-13a	*Saccoglossus kowalevskii*	Sko_Hox11-13a	Hemichordata	Enteropneusta

ABK00023	Hox11-13b	*Saccoglossus kowalevskii*	Sko_Hox11-13b	Hemichordata	Enteropneusta
AAP79288	Hox11-13c	*Saccoglossus kowalevskii*	Sko_Hox11-13c	Hemichordata	Enteropneusta
ABK00018	Hox2	*Saccoglossus kowalevskii*	Sko_Hox2	Hemichordata	Enteropneusta
AAP79286	Hox3	*Saccoglossus kowalevskii*	Sko_Hox3	Hemichordata	Enteropneusta
AAP79297	Hox4	*Saccoglossus kowalevskii*	Sko_Hox4	Hemichordata	Enteropneusta
ABK00019	Hox5	*Saccoglossus kowalevskii*	Sko_Hox5	Hemichordata	Enteropneusta
ABK00020	Hox6	*Saccoglossus kowalevskii*	Sko_Hox6	Hemichordata	Enteropneusta
AAP79287	Hox7	*Saccoglossus kowalevskii*	Sko_Hox7	Hemichordata	Enteropneusta
ABK00021	Hox9-10	*Saccoglossus kowalevskii*	Sko_Hox9-10	Hemichordata	Enteropneusta
Currie et al. 2016[2]	Hox1	*Schmidtea mediterranea*	Sme_Hox1	Platyhelminthes	Rhabditophora
Currie et al. 2016[2]	Hox2-3a	*Schmidtea mediterranea*	Sme_Hox2-3a	Platyhelminthes	Rhabditophora
Currie et al. 2016[2]	Hox4a	*Schmidtea mediterranea*	Sme_Hox4a	Platyhelminthes	Rhabditophora
Currie et al. 2016[2]	Lox5a	*Schmidtea mediterranea*	Sme_Lox5a	Platyhelminthes	Rhabditophora
Currie et al. 2016[2]	Post2a	*Schmidtea mediterranea*	Sme_Post2a	Platyhelminthes	Rhabditophora
Currie et al. 2016[2]	Post2b	*Schmidtea mediterranea*	Sme_Post2b	Platyhelminthes	Rhabditophora
Currie et al. 2016[2]	Post2c	*Schmidtea mediterranea*	Sme_Post2c	Platyhelminthes	Rhabditophora
Currie et al. 2016[2]	Post2d	*Schmidtea mediterranea*	Sme_Post2d	Platyhelminthes	Rhabditophora
AAN11405	Central	*Symsagittifera roscoffensis*	Sro_Central	Xenacoelomorpha	
ACM69150.1	Hox1	*Symsagittifera roscoffensis*	Sro_Hox1	Xenacoelomorpha	
AAN11404	Lab	*Symsagittifera roscoffensis*	Sro_Lab	Xenacoelomorpha	
AAN11406	Post	*Symsagittifera roscoffensis*	Sro_Post	Xenacoelomorpha	
ADJ18233.1	Hox2	*Steromphala varia*	Sva_Hox2	Mollusca	Gastropoda
ADJ18232.1	Hox3	*Steromphala varia*	Sva_Hox3	Mollusca	Gastropoda
ACX84672.1	Hox4	*Steromphala varia*	Sva_Hox4	Mollusca	Gastropoda
ADJ18234.1	Hox5	*Steromphala varia*	Sva_Hox5	Mollusca	Gastropoda
ADJ18236.1	Hox7	*Steromphala varia*	Sva_Hox7	Mollusca	Gastropoda
ACX84671.1	HoxA	*Steromphala varia*	Sva_HoxA	Mollusca	Gastropoda
ADJ18238.1	Lox2	*Steromphala varia*	Sva_Lox2	Mollusca	Gastropoda
ADJ18237.1	Lox4	*Steromphala varia*	Sva_Lox4	Mollusca	Gastropoda
ADJ18235.1	Lox5	*Steromphala varia*	Sva_Lox5	Mollusca	Gastropoda
ACX84673.1	Post1	*Steromphala varia*	Sva_Post1	Mollusca	Gastropoda
AAB70263	AbdA	*Tribolium castaneum*	Tca_AbdA	Arthropoda	Insecta
AAF36721	AbdB	*Tribolium castaneum*	Tca_AbdB	Arthropoda	Insecta
EEZ99250	Antp	*Tribolium castaneum*	Tca_Antp	Arthropoda	Insecta
AAK16423	Dfd	*Tribolium castaneum*	Tca_Dfd	Arthropoda	Insecta
NP_001034538.1	Evx	*Tribolium castaneum*	Tca_Evx	Arthropoda	Insecta
AAK16421	Ftz	*Tribolium castaneum*	Tca_Ftz	Arthropoda	Insecta
AAF64148	Lab	*Tribolium castaneum*	Tca_Lab	Arthropoda	Insecta
NP_001107807.1	Mxp	*Tribolium castaneum*	Tca_Mxp	Arthropoda	Insecta
AAK16422	Scr	*Tribolium castaneum*	Tca_Scr	Arthropoda	Insecta
NP_001034497	Ubx	*Tribolium castaneum*	Tca_Ubx	Arthropoda	Insecta
NP_001036813	Zen1	*Tribolium castaneum*	Tca_Zen1	Arthropoda	Insecta
AAK16425.1	Zen2	*Tribolium castaneum*	Tca_Zen2	Arthropoda	Insecta
AAW72807	Lox2	*Urechis unicinctus*	Uun_Lox2	Annelida	Sedentaria
Otx **Substitution model: VT + G**					
**Accession**	**Gene ID**	***Species***	**Sample ID**	**Phylum**	**SubGroup (Subphylum/ Class/Clade)**

ABK41270.1	OtxA	*Acropora millepora*	Ami_OtxA	Cnidaria	
ABK41271.1	OtxB	*Acropora millepora*	Ami_OtxB	Cnidaria	
AAC00193	Otx	*Branchiostoma floridae*	Bfl_Otx	Chordata	Cephalochordata
NP_001027662	Otx	*Ciona intestinalis*	Cin_Otx	Chordata	Tunicata
ALJ33544.1	Otp	*Clytia hemisphaerica*	Che_Otp	Cnidaria	Hydrozoa
P22810.2	Otd	*Drosophila melanogaster*	Dme_Otd	Arthropoda	Insecta
EDW58830.2	Otp	*Drosophila virilis*	Dvi_Otp	Arthropoda	Insecta
AMR72028.1	Otx	*Lineus ruber*	Lru_Otx	Nemertea	
ARJ36943.1	Otx	*Membranipora membranacea*	Mme_Otx	Bryozoa	
NP_035153	Otx1	*Mus musculus*	Mmu_Otx1	Chordata	Vertebrata
NP_659090	Otx2	*Mus musculus*	Mmu_Otx2	Chordata	Vertebrata
NP_035151.1	Otp	*Mus musculus*	Mmu_Otp	Chordata	Vertebrata
AHY88455.1	Otx	*Novocrania anomala*	Nan_Otx	Brachiopoda	
AAM33144.1	Otx	*Patella vulgata*	Pvu_Otx	Mollusca	Gastropoda
AAP32748	Otxβ-a	*Patiria miniata*	Pmi_Otxβ-a	Echinodermata	Asteroidea
ABR68849.1	Otp	*Platynereis dumerilii*	Pdu_Otp	Annelida	
AFY12008.1	Otx	*Priapulus caudatus*	Pca_Otx	Priapulida	
BAA89013	Otx	*Ptychodera flava*	Pfl_Otx	Hemichordata	Enteropneusta
Six **Substitution model: LG + G**					
**Accession**	**Gene ID**	***Species***	**Sample ID**	**Phylum**	**SubGroup (Subphylum/ Class/Clade)**
KAK6999249.1	Six4	*Biomphalaria glabrata*	Bgl_Six4	Mollusca	Gastropoda
KAF6038190.1	Six6	*Bugula neritina*	Bne_Six6	Bryozoa	
UYV63952.1	Six2	*Cordylochernes scorpioides*	Csc_Six2	Arthropoda	Chelicerata
JAK25541.1	Six4	*Daphnia magna*	Dma_Six4	Arthropoda	
P40427.1	Exd	*Drosophila melanogaster*	Dme_Pbx	Arthropoda	Insecta
AAF63760.1	myotonix	*Drosophila melanogaster*	Dme_Six5	Arthropoda	Insecta
NP_524695	Optix	*Drosophila melanogaster*	Dme_Optix	Arthropoda	Insecta
NP_476733	Sine oculis	*Drosophila melanogaster*	Dme_So	Arthropoda	Insecta
GFS24247.1	Exd	*Elysia marginata*	Ema_Exd	Mollusca	Gastropoda
WGZ76181.1	Six1	*Hofstenia miamia*	Hmi_Six1	Xenacoelomorpha	
KAI4083748.1	Pbx	*Homo sapiens*	Hsa_Pbx	Chordata	Vertebrata
KAI0215952.1	Six4	*Lamellibrachia satsuma*	Lsa_Six4	Annelida	
XP_064624184.1	Six1-like	*Lineus longissimus*	Llo_Six1	Nemertea	
XP_013418710.1	Six6	*Lingula anatina*	Lan_Six6	Brachiopoda	
NP_033215	Six1	*Mus musculus*	Mmu_Six1	Chordata	Vertebrata
NP_035510	Six2	*Mus musculus*	Mmu_Six2	Chordata	Vertebrata
NP_035511	Six3	*Mus musculus*	Mmu_Six3	Chordata	Vertebrata
NP_035512	Six4	*Mus musculus*	Mmu_Six4	Chordata	Vertebrata
NP_035513	Six5	*Mus musculus*	Mmu_Six5	Chordata	Vertebrata
NP_035514	Six6	*Mus musculus*	Mmu_Six6	Chordata	Vertebrata
XP_032228423.1	Six3	*Nematostella vectensis*	Nve_Six3	Cnidaria	
CAI9729785.1	Six6	*Octopus vulgaris*	Ovu_Six6	Mollusca	Cephalopoda
AMZ00043.1	Exd	*Platynereis dumerilii*	Pdu_Exd	Annelida	
CAR66435.1	Six3	*Platynereis dumerilii*	Pdu_Six3	Annelida	
ACG76359.1	Pbx	*Saccoglossus kowalevskii*	Sko_Pbx	Hemichordata	Enteropneusta
AAP79281	Six3	*Saccoglossus kowalevskii*	Sko_Six3	Hemichordata	Enteropneusta
XP_012797748.1	Six6	*Schistosoma haematobium*	Sha_Six6	Platyhelminthes	

XP_781696	Six3	*Strongylocentrotus purpuratus*	Spu_Six3	Echinodermata	Echinoidea
XP_781616	Six4	*Strongylocentrotus purpuratus*	Spu_Six4	Echinodermata	Echinoidea
AJV21321.1]	Six1/2	*Terebratalia transversa*	Ttr_Six1-2	Brachiopoda	
KRZ36758.1	Six5	*Trichinella pseudospiralis*	Tps_Six5	Nematoda	
XP_067941307.1	Six1-like	*Watersipora subatra*	Wsu_Six1	Bryozoa	

Supplementary Table 2. Summary of transcriptomic datasets used for Hox gene screening in chaetognaths and rotifer

Species	Identifier	Accession Number	No. of Reads
Paraspadella gotoi	Pgo	SRR8149062	45,906,643
Eukrohnia hamata	Eha	SRR7754745	32,806,427
Krohnitta subtilis	Ksu	SRR7754744	34,018,209
Pterosagitta draco	Pdr	SRR7754743	26,198,377
Sagitta elegans	Sel	SRR7754742	41,790,715
Rotaria rotatoria	Rro	ERR454505)	46,904,522

Supplementary Table 3. Primer sequences and PCR annealing temperature used for the isolation of the *Spadella cephaloptera* gene sequences.

Gene	Forward Primer	Sequence (5'-3')	Reverse primer	Sequence (5'-3')	T_A (°C)	Amplification Method	Riboprobe Length (bp)	Transcript Length (bp)
Sce-hox1	Sce-hox1-01F	CAAGTTCCCAACTACAAGTGGATGCACCTC	Sce-hox1-01R	TAATACGACTCACTATAGGGGAGAATAGCGTCGGCGAGCGGATTG	61	PCR-T7	658	2516
Sce-hox3	Sce-hox3-05F	GCCCATATGAACATGCTCACCG	Sce-hox3-05R	TAATACGACTCACTATAGGGCTTTCGTCGTCATCATCGTCGC	60	PCR-T7	920	3319
Sce-hox4	Sce-hox4-03F	CCTGGAACTGGAGAAGGAGTTTC	Sce-hox4-03R	TAATACGACTCACTATAGGGTCCATGTCGTTGTCCATGGTG	59	PCR-T7	459	760
Sce-hox5	Sce-hox5-02F	AGGAAGGTTCACATGAGTCACG	Sce-hox5-02R	TAATACGACTCACTATAGGGCAAACGTGTGAGGAGAGTTGTG	61	PCR-T7	809	2664
Sce-hox6	Sce-hox6-01F	GAACCACCGGAGATTTGGGCATCGATC	Sce-hox6-01R	TAATACGACTCACTATAGGGGTCGTTTGTTTCGTTGTTTATATATCCCTCCGGTTG	61	PCR-T7	576	2664
Sce-hox7	Sce-hox7-01F	CAAACGCCCATCTATCCTTGGATG	Sce-hox7-01R	CATTTCCCCATTCACCCATGACTTC	61	cloning	464	1371
Sce-hox8	Sce-hox8-01F	GTGCTCGGCAACTCCTACTCATCTCATC	Sce-hox8-01R	TAATACGACTCACTATAGGGGAGTTGTCGAGTTTGTGGATGGCGTC	61	PCR-T7	626	1245
Sce-postA	Sce-postA-01F	GATTCCATCAACACCACGCAGC	Sce-postA-01R	TAATACGACTCACTATAGGGAATGCGTTGAAGTCGGCGATC	60	PCR-T7	572	1156
Sce-postB	Sce-postB-03F	GGCCTCGACTTCAACAAGATTGAC	Sce-postB-01R	TAATACGACTCACTATAGGGGATGAAGGAGCTTGGTGGGATGG	55	PCR-T7	535	1091
Sce-six3/6	Sce-six3/6-01F	CACGCGCACTCGACGCCTTTC	Sce-six3/6-01R	TAATACGACTCACTATAGGGGATGATGTTTATGCATCCTGTTTTTCGCGGATG	60	PCR-T7	828	979

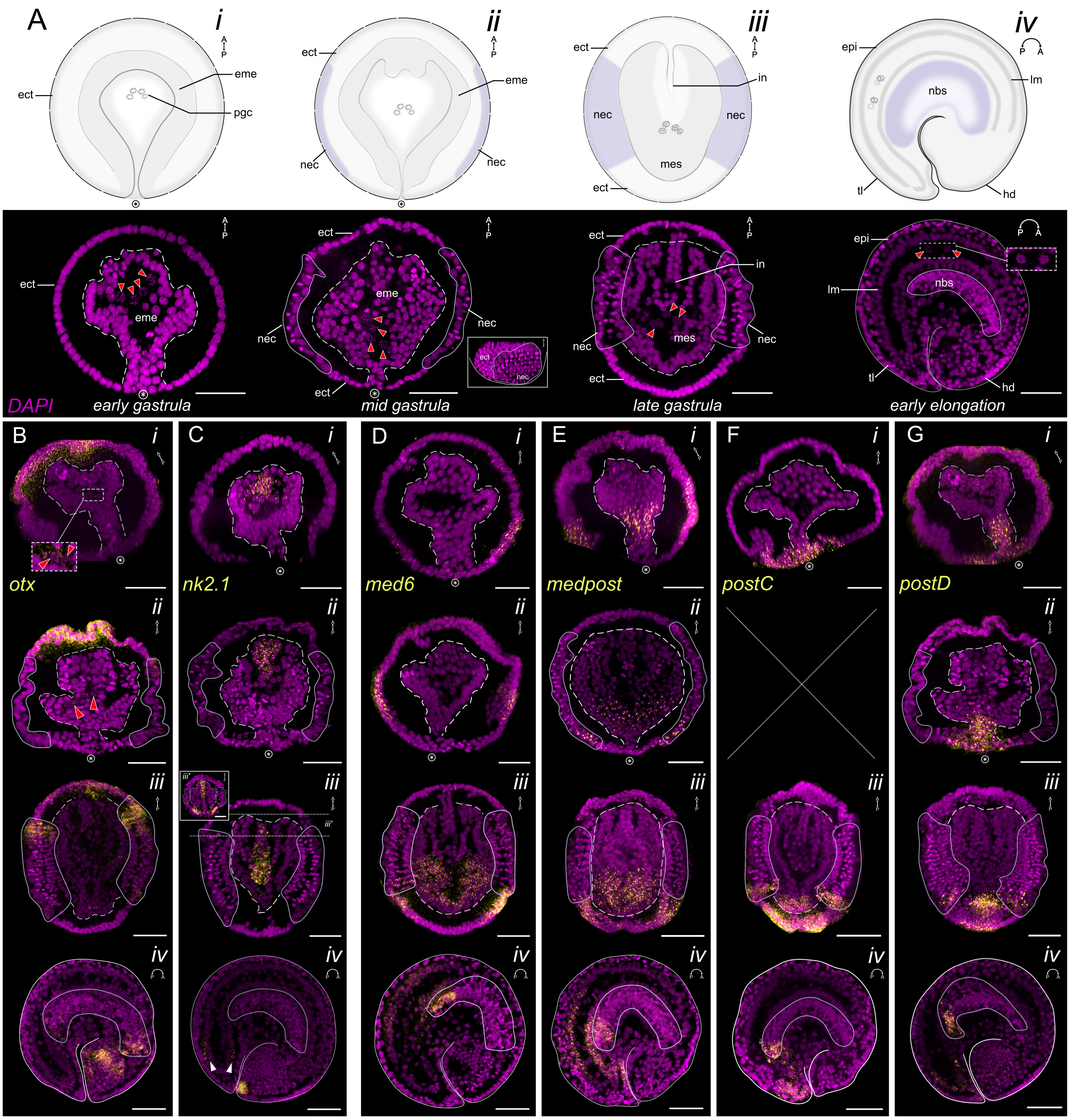

A
i
ii
iii
iv
ect
eme
pgc
nec
in
mes
epi
lm
nbs
tl
hd
DAPI
early gastrula
mid gastrula
late gastrula
early elongation
B
C
D
E
F
G
otx
nk2.1
med6
medpost
postC
postD

Supplementary Figure 1. Embryonic stages and expression patterns of anterior patterning and Hox genes in *Spadella cephaloptera*. Gene transcripts (yellow) are visualized with AlexaFluor-647, and cell nuclei are counterstained with DAPI (purple). (A, top row) Schematic representations of early (i), mid (ii), and late gastrula (iii) in dorsal view, and early elongation stage (iv) in lateral view. (A, bottom row) Corresponding DAPI-stained optical sections. Inset in panel *ii* shows the neuroectoderm (*nec*) in lateral view, with a white outline marking the embryo. Encircled asterisk indicates the position of the blastopore. Primordial germ cells (*pgc*) are marked with red arrowheads. In panel *iv*, PGCs are shown at higher magnification and contrast in the inset. (B – G) Expression patterns of *Sce-otx* (B), *Sce-nk2.1* (C), *Sce-med6* (D), *Sce-medpost* (E), *Sce-postC* (F), *Sce-postD* (G) across embryonic stages (panels *i – iv*). Gastrula stages (*i – iii*) are in dorsal view and early elongation (panel *iv*) is in lateral view. No expression data are available for *Sce-postC* at the mid gastrula stage. Inset (*iii'*) in panel *Ciii* shows a transverse section through the anterior portion of the embryo (dashed line) with expression in the ventral ectoderm. Purple outlines demarcate the neuroectoderm (*ii*, *iii*) and the developing ventral nerve center (*iv*). Scale bars: 50 µm. Orientation is indicated in the upper right corner of each panel. ect, ectoderm; eme, endomesoderm; epi, epidermis; hd, head bud; in, intestine; lm, longitudinal muscle progenitor cells; mes, mesoderm; nbs, neuroblast of the developing VNC; nec, neuroectoderm; tl, tail bud

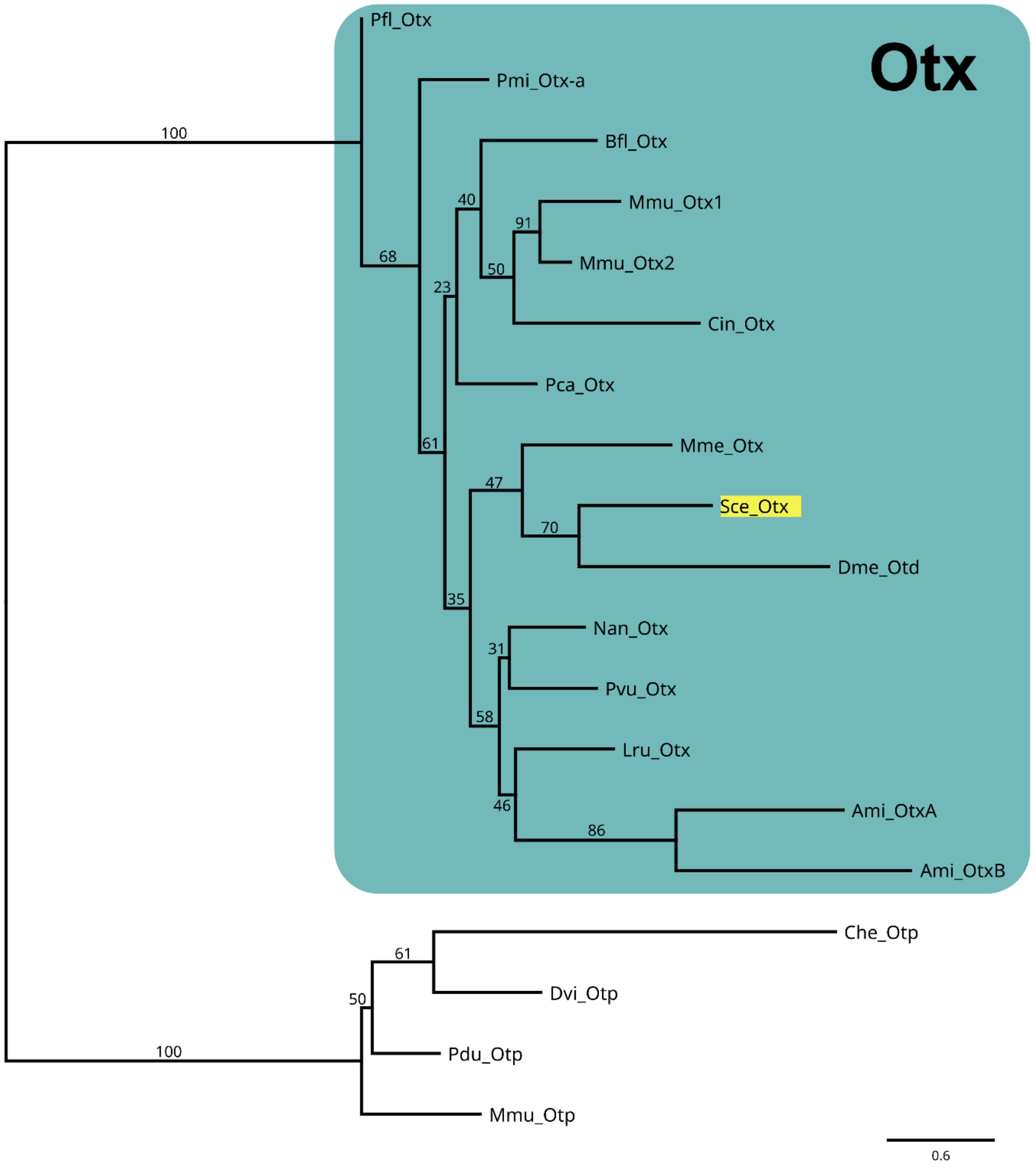

Supplementary Figure 2. Orthology analysis including the deduced amino acid sequence of *otx* of *Spadella cephaloptera***.** The phylogenetic tree of *otx* genes is based on bilaterian protein sequences obtained from published literature and BLAST searches of the NCBI GenBank. The tree was generated using Maximum Likelihood analysis implemented in IQTREE with the following configurations: LG + G was selected based on ModelFinder and ultrafast bootstrap set to 1000. The support values of branches indicate maximum likelihood bootstrap values. The tree is rooted with the deduced amino acid sequence of *otp* (*orthopedia*). The Otx group is highlighted in the teal box and Sce-Otx in yellow. Species abbreviations are in Supplementary Table 1.

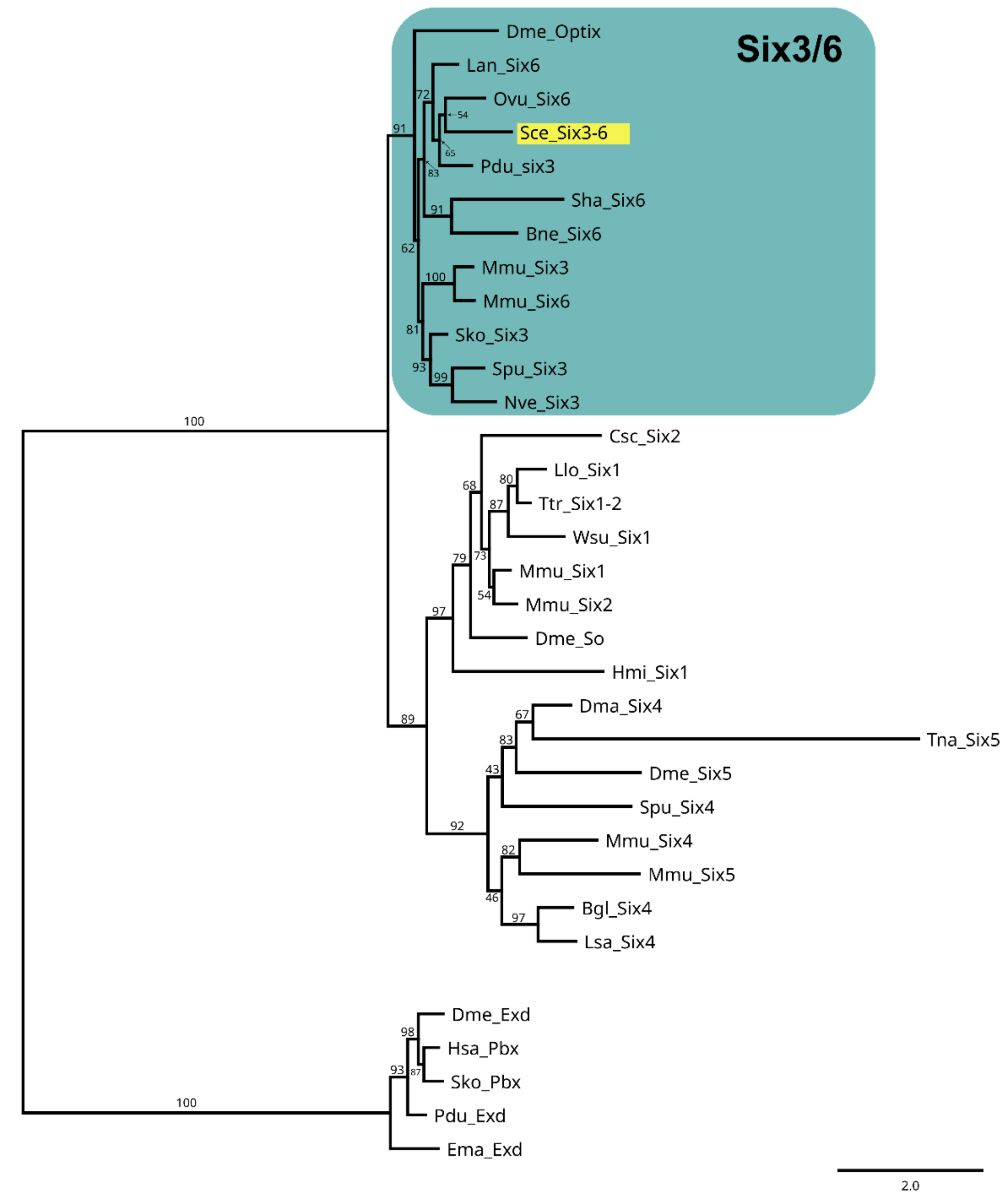

Supplementary Figure 3. Orthology analysis including the deduced amino acid sequence of *six3/6* of *Spadella cephaloptera***.** The phylogenetic tree of *six* genes is based on bilaterian protein sequences obtained from published literature and BLAST searches of the NCBI GenBank. The tree was generated using Maximum Likelihood analysis implemented in IQTREE with the following configurations: LG + G was selected based on ModelFinder and ultrafast bootstrap set to 1000. The support values of branches indicate maximum likelihood bootstrap values. The tree is rooted with the deduced amino acid sequence of *pbx* (*PBX Homeobox 1*)/*exd* (*extradenticle*) genes as an outgroup. The Six3/6 group is highlighted in the teal box and Sce-six3/6 in yellow. Species abbreviations are in Supplementary Table 1.

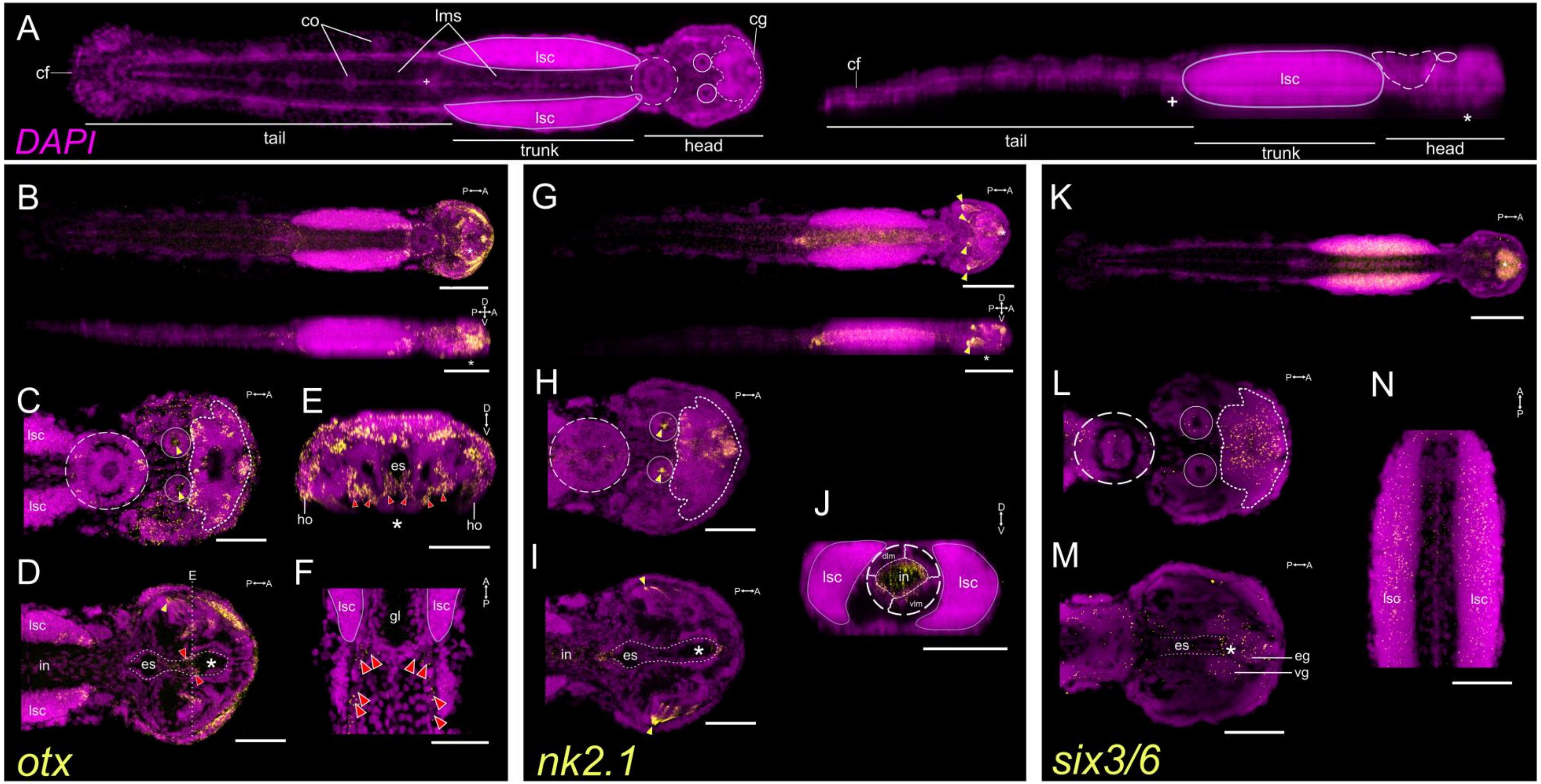

A
co
lms
cg
cf
lsc
lsc
tail
trunk
head
DAPI
cf
lsc
tail
trunk
head
B
C
lsc
lsc
E
es
ho
ho
D
E
lsc
in
es
lsc
F
lsc
lsc
gl
otx
G
H
I
in
es
J
lsc
dlm
in
vlm
lsc
nk2.1
K
L
N
M
es
eg
vg
lsc
lsc
six3/6

Supplementary Figure 4. Expression patterns of anterior patterning genes in the early juvenile (7-10 dph) of *Spadella cephaloptera*. Gene transcripts (yellow) are visualized with AlexaFluor647 (B – J) or AP-Fast Blue (K – N), and cell nuclei are counterstained with DAPI (purple). (A) General morphology of an early juvenile shown in dorsal (left) and lateral (right) views from DAPI-based maximum intensity projections. Eye locations are encircled and the corona ciliata is outlined with dashed lines. The asterisk and crosshair mark the positions of the mouth and anal opening, respectively. (B – F) *Sce-otx* expression pattern. (B) Dorsal and lateral maximum projections. (C) Dorsal optical section showing expression in the dorsal head structures, including the corona ciliata (dashed circle), eyes (solid circles), and cerebral ganglion (dotted outline). Yellow arrowheads indicate autofluorescent structures. (D) Dorsal section along the intestine (in), and (E) transverse section through the posterior cerebral ganglion. *Sce-otx* signal is detected in the perioral epidermis (red arrowheads) between the mouth and esophagus (D), and at the base of the head (E). (F) Higher magnification of dividing germ cells (dotted outlines), showing female (top) and male (bottom) germ cells. (G – J) *Sce-nk2.1* expression pattern. (G) Dorsal and lateral maximum projections. (H) Dorsal optical section of the head showing expression in the medial region of the cerebral ganglion. (I) Dorsal view showing expression in the posterior esophagus and anterior mouth. (J) Transverse section of the trunk showing broad *Sce-nk2.1* expression in the intestine. (K – N) *Sce-six3/6* expression pattern. (K) Dorsal and lateral maximum projections. (L) Dorsal view of the head. (M) Dorsal section showing expression in the esophageal ganglia (eg) and vestibular ganglia (vg). (N) Dorsal profile of the trunk showing scattered *Sce-six3/6* signal. Scale bars: 50 µm, except *B*, *G*, and *K* (100 µm). cf, caudal fin; cg, cerebral ganglion; co, ciliary tuft/fence organ; dlm, dorsal longitudinal muscle; es, esophagus; ho, hood; in, intestine; lms: longitudinal mucles somata; lsc, lateral somata clusters; vlm, ventral longitudinal muscle

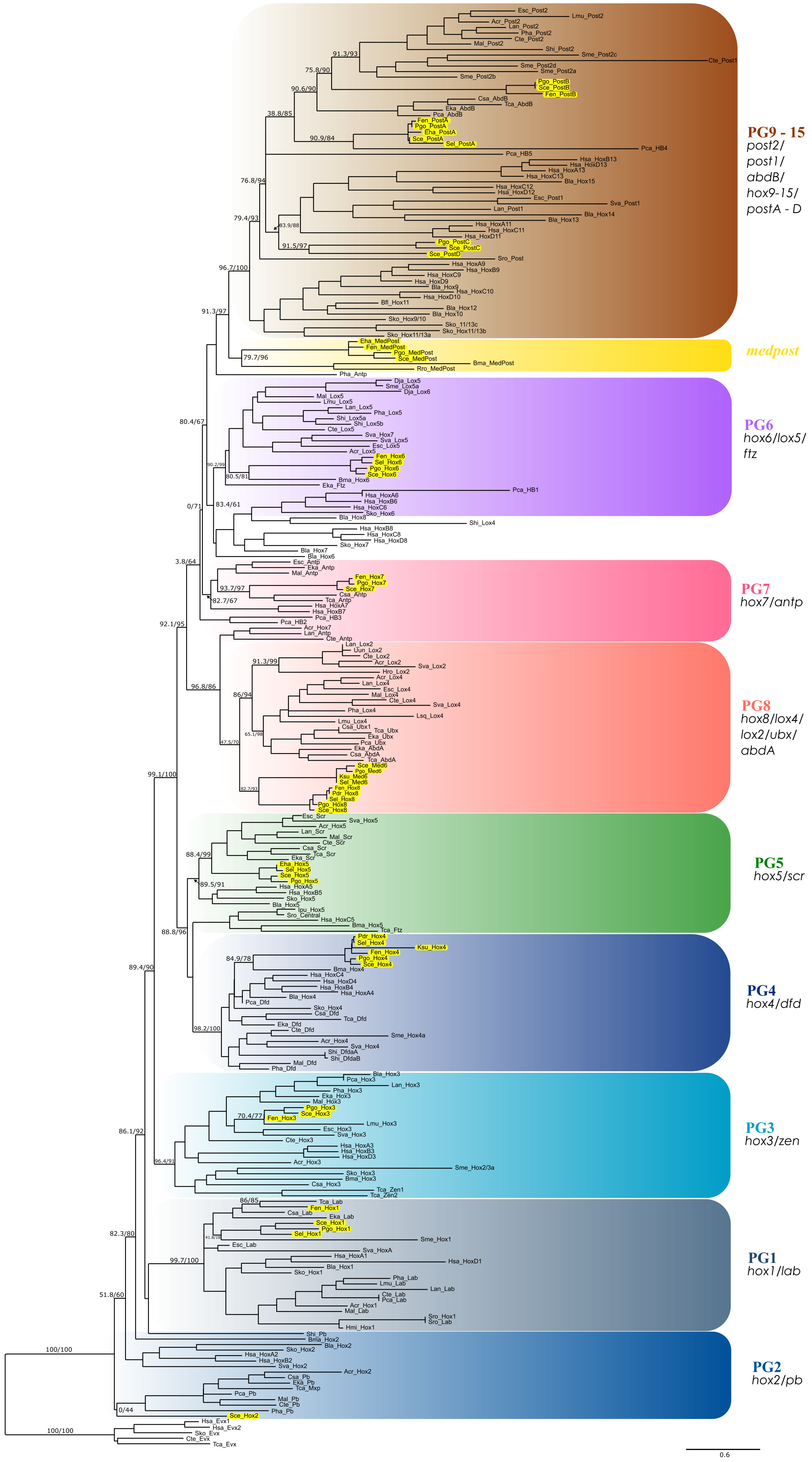
PG9 - 15
post2/
post1/
abdB/
hox9-15/
postA - D
medpost
PG6
hox6/lox5/
ftz
PG7
hox7/antp
PG8
hox8/lox4/
lox2/ubx/
abdA
PG5
hox5/scr
PG4
hox4/dfd
PG3
hox3/zen
PG1
hox1/lab
PG2
hox2/pb
Esc_Post2
Lmu_Post2
Acr_Post2
Lan_Post2
Pha_Post2
Cte_Post2
Mal_Post2
Shi_Post2
Sme_Post2c
Cte_Post1
Sme_Post2d
Sme_Post2a
Sme_Post2b
Pgo_PostB
Sce_PostB
Fen_PostB
Csa_AbdB
Tca_AbdB
Eka_AbdB
Pca_AbdB
Fen_PostA
Pgo_PostA
Eha_PostA
Sce_PostA
Sel_PostA
Pca_HB5
Pca_HB4
Hsa_HoxB13
Hsa_HoxD13
Hsa_HoxA13
Hsa_HoxC13
Bla_Hox15
Hsa_HoxC12
Hsa_HoxD12
Esc_Post1
Sva_Post1
Lan_Post1
Bla_Hox14
Bla_Hox13
Hsa_HoxA11
Hsa_HoxC11
Hsa_HoxD11
Pgo_PostC
Sce_PostC
Sce_PostD
Sro_Post
Hsa_HoxA9
Hsa_HoxB9
Hsa_HoxC9
Hsa_HoxD9
Bla_Hox9
Hsa_HoxC10
Hsa_HoxD10
Bfl_Hox11
Bla_Hox12
Bla_Hox10
Sko_Hox9/10
Sko_11/13c
Sko_Hox11/13b
Sko_Hox11/13a
Eha_MedPost
Fen_MedPost
Pgo_MedPost
Sce_MedPost
Bma_MedPost
Rro_MedPost
Pha_Antp
Dja_Lox5
Sme_Lox5a
Dja_Lox6
Mal_Lox5
Lmu_Lox5
Lan_Lox5
Pha_Lox5
Shi_Lox5a
Shi_Lox5b
Cte_Lox5
Sva_Hox7
Sva_Lox5
Esc_Lox5
Acr_Lox5
Fen_Hox6
Sel_Hox6
Pgo_Hox6
Sce_Hox6
Bma_Hox6
Eka_Ftz
Pca_HB1
Hsa_HoxA6
Hsa_HoxB6
Hsa_HoxC6
Sko_Hox6
Bla_Hox8
Shi_Lox4
Hsa_HoxB8
Hsa_HoxC8
Hsa_HoxD8
Sko_Hox7
Bla_Hox7
Bla_Hox6
Esc_Antp
Eka_Antp
Mal_Antp
Fen_Hox7
Pgo_Hox7
Sce_Hox7
Csa_Antp
Tca_Antp
Hsa_HoxA7
Hsa_HoxB7
Pca_HB3
Pca_HB2
Acr_Hox7
Lan_Antp
Cte_Antp
Lan_Lox2
Uun_Lox2
Cte_Lox2
Acr_Lox2
Hro_Lox2
Sva_Lox2
Acr_Lox4
Lan_Lox4
Esc_Lox4
Mal_Lox4
Cte_Lox4
Sva_Lox4
Pha_Lox4
Lsq_Lox4
Lmu_Lox4
Csa_Ubx1
Tca_Ubx
Eka_Ubx
Pca_Ubx
Eka_AbdA
Csa_AbdA
Tca_AbdA
Sce_Med6
Pgo_Med6
Ksu_Med6
Sel_Med6
Fen_Hox8
Pdr_Hox8
Sel_Hox8
Pgo_Hox8
Sce_Hox8
Esc_Scr
Sva_Hox5
Acr_Hox5
Lan_Scr
Mal_Scr
Cte_Scr
Csa_Scr
Tca_Scr
Eka_Scr
Eha_Hox5
Sel_Hox5
Sce_Hox5
Pgo_Hox5
Hsa_HoxA5
Hsa_HoxB5
Sko_Hox5
Bla_Hox5
Ipu_Hox5
Sro_Central
Hsa_HoxC5
Bma_Hox5
Tca_Ftz
Pdr_Hox4
Sel_Hox4
Ksu_Hox4
Fen_Hox4
Pgo_Hox4
Sce_Hox4
Bma_Hox4
Hsa_HoxC4
Hsa_HoxD4
Hsa_HoxB4
Hsa_HoxA4
Bla_Hox4
Pca_Dfd
Sko_Hox4
Csa_Dfd
Tca_Dfd
Eka_Dfd
Cte_Dfd
Sme_Hox4a
Acr_Hox4
Sva_Hox4
Shi_DfdaA
Shi_DfdaB
Mal_Dfd
Pha_Dfd
Bla_Hox3
Pca_Hox3
Lan_Hox3
Pha_Hox3
Eka_Hox3
Mal_Hox3
Pgo_Hox3
Sce_Hox3
Fen_Hox3
Lmu_Hox3
Esc_Hox3
Sva_Hox3
Cte_Hox3
Hsa_HoxA3
Hsa_HoxB3
Hsa_HoxD3
Acr_Hox3
Sme_Hox2/3a
Sko_Hox3
Bma_Hox3
Csa_Hox3
Tca_Zen1
Tca_Zen2
Tca_Lab
Fen_Hox1
Csa_Lab
Eka_Lab
Sce_Hox1
Pgo_Hox1
Sel_Hox1
Sme_Hox1
Esc_Lab
Sva_HoxA
Hsa_HoxA1
Hsa_HoxD1
Bla_Hox1
Sko_Hox1
Pha_Lab
Lmu_Lab
Lan_Lab
Cte_Lab
Pca_Lab
Acr_Hox1
Mal_Lab
Sro_Hox1
Sro_Lab
Hmi_Hox1
Shi_Pb
Bma_Hox2
Bla_Hox2
Sko_Hox2
Hsa_HoxA2
Hsa_HoxB2
Sva_Hox2
Acr_Hox2
Csa_Pb
Eka_Pb
Tca_Mxp
Pca_Pb
Mal_Pb
Cte_Pb
Pha_Pb
Sce_Hox2
Hsa_Evx1
Hsa_Evx2
Sko_Evx
Cte_Evx
Tca_Evx
91.3/93
75.8/90
90.6/90
38.8/85
90.9/84
76.8/94
79.4/93
83.9/88
91.5/97
96.7/100
91.3/97
79.7/96
80.4/67
90.2/99
80.5/81
0/71
83.4/61
3.8/64
93.7/97
82.7/67
92.1/95
91.3/99
96.8/86
86/94
65.1/98
47.5/70
82.7/93
99.1/100
88.4/99
89.5/91
88.8/96
84.9/78
89.4/90
98.2/100
70.4/77
86.1/92
96.4/91
86/85
82.3/80
99.7/100
51.8/60
100/100
0/44
100/100
0.6

Supplementary Figure 5. Orthology analysis of chaetognath Hox genes. A phylogenetic tree based on 261 amino acid sequences was generated using Maximum Likelihood analysis and SH-aLRT test implemented in IQTREE. The support values of branches indicate SH-aLRT and ultrafast boostrap support (SS% / UFBS%). The tree is rooted with *even-skipped* (*evx*) sequences from various bilaterian species. Support values are shown only for key nodes. Paralogous groups (PGs) are highlighted with different box colors, and chaetognath Hox genes are highlighted in yellow. Species abbreviations are listed in Table S1.

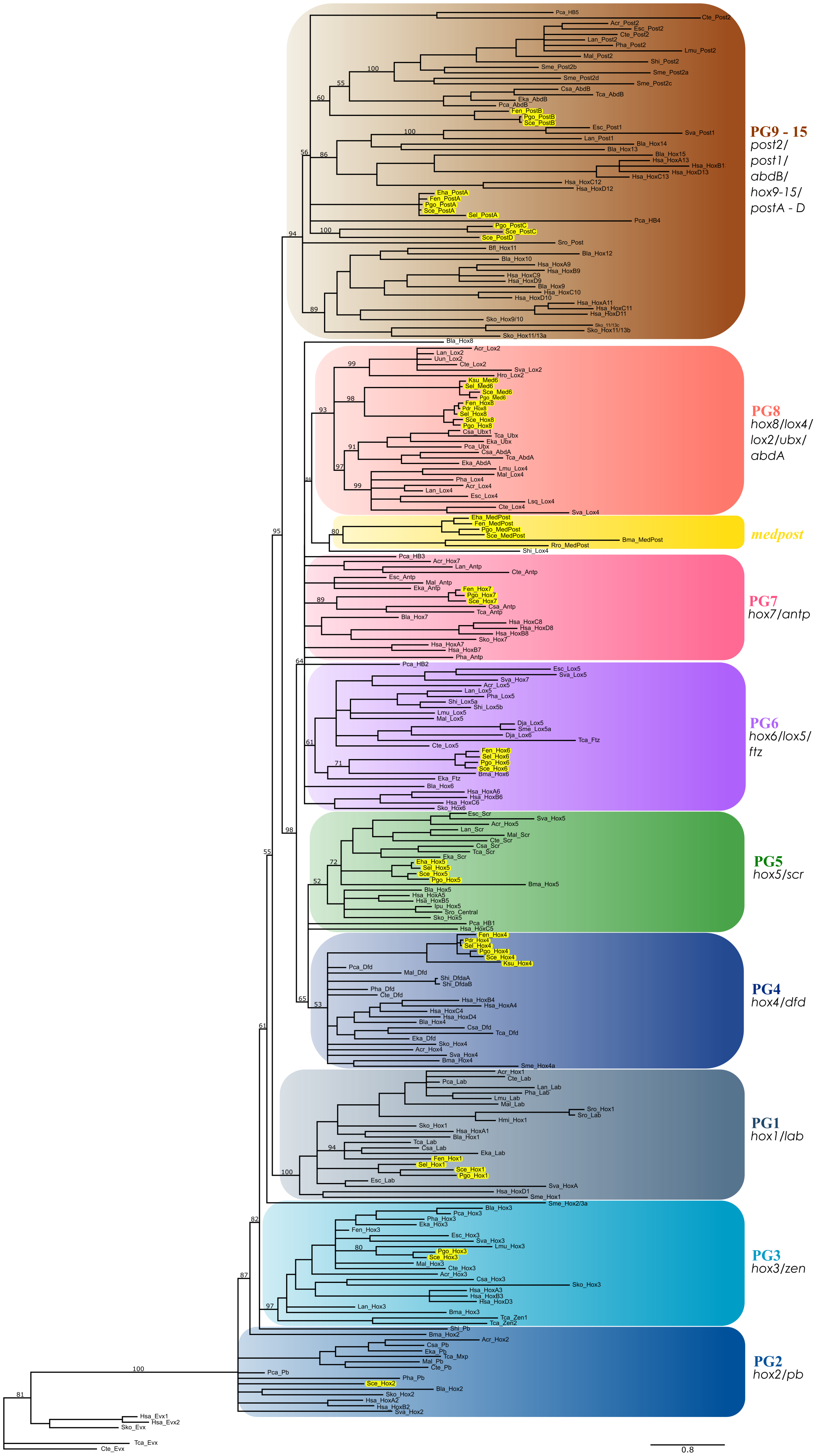

PG9 - 15
post2/
post1/
abdB/
hox9-15/
postA - D
PG8
hox8/lox4/
lox2/ubx/
abdA
medpost
PG7
hox7/antp
PG6
hox6/lox5/
ftz
PG5
hox5/scr
PG4
hox4/dfd
PG1
hox1/lab
PG3
hox3/zen
PG2
hox2/pb
0.8

Supplementary Figure 6. Orthology analysis of chaetognath Hox genes. A phylogenetic tree based on 261 amino acid sequences was generated using Bayesian inference implemented in MrBayes. Branch support values represent posterior probabilities. The tree is rooted with the deduced amino acid sequences of *even-skipped* (*evx*) from various bilaterian species. Support values are shown only for key nodes. Paralogous groups (PGs) are highlighted with different box colors, and chaetognath Hox genes are highlighted in yellow. Species abbreviations are listed in Table S1.

```
                  10        20        30        40        50        60
          ----:----|----:----|----:----|----:----|----:----|----:----|----:--
Consensus PRRLRTAYTNTQLLELEKEFHFNKYLCRPRRIEIAASLDLTERQVKVWFQNRRMKHKRQTQKKKXXD
Acr_Hox2  T......................................................Y...S.IQ.HG-
Sva_Hox2  S......................................................Y...S.SGRSKS
Mal_Pb    ..........S............................................Y...SG------
Cte_Pb    .......................................................F....G.GSGNS
Csa_Pb    ............................................................SVM.DD.
Ctu_Pb    -------------------............................--------------------
Tca_Mxp   ............................................................LG.QGD.
Eka_Pb    ...........................................................NLG.GAE.
Pca_Pb    --------------------....................S....----------------------
Sce_Hox2  ..........................................................HSLQTGPGA
Bpl_Hox2  H..I......N.........FN.........V...SN.S.......I..........KERSH..NRK
Bla_Hox2  S.....VF.............Y...V.K...K...SF...N.....I.......RQ..RDT.SRSEI
Sko_Hox2  H..V...F.............Y.............SM...S..................IM.AAVSG
Hsa_HoxA2 S..............................V....L........................C.ENQN
Hsa_HoxB2 A..............................V....L........................HREPP.
```

Supplementary Figure 7. Hox domain alignment of bilaterian PG2**.** Diagnostic residues for the deduced amino acid sequence of *Hox2*/*proboscipedia (pb)* are highlighted in yellow. Chaetognatha-Gnathifera sequences are in dark blue and *Spadella cephaloptera* sequences are in **bold** letters. Species abbreviations are in Supplementary Table 1.

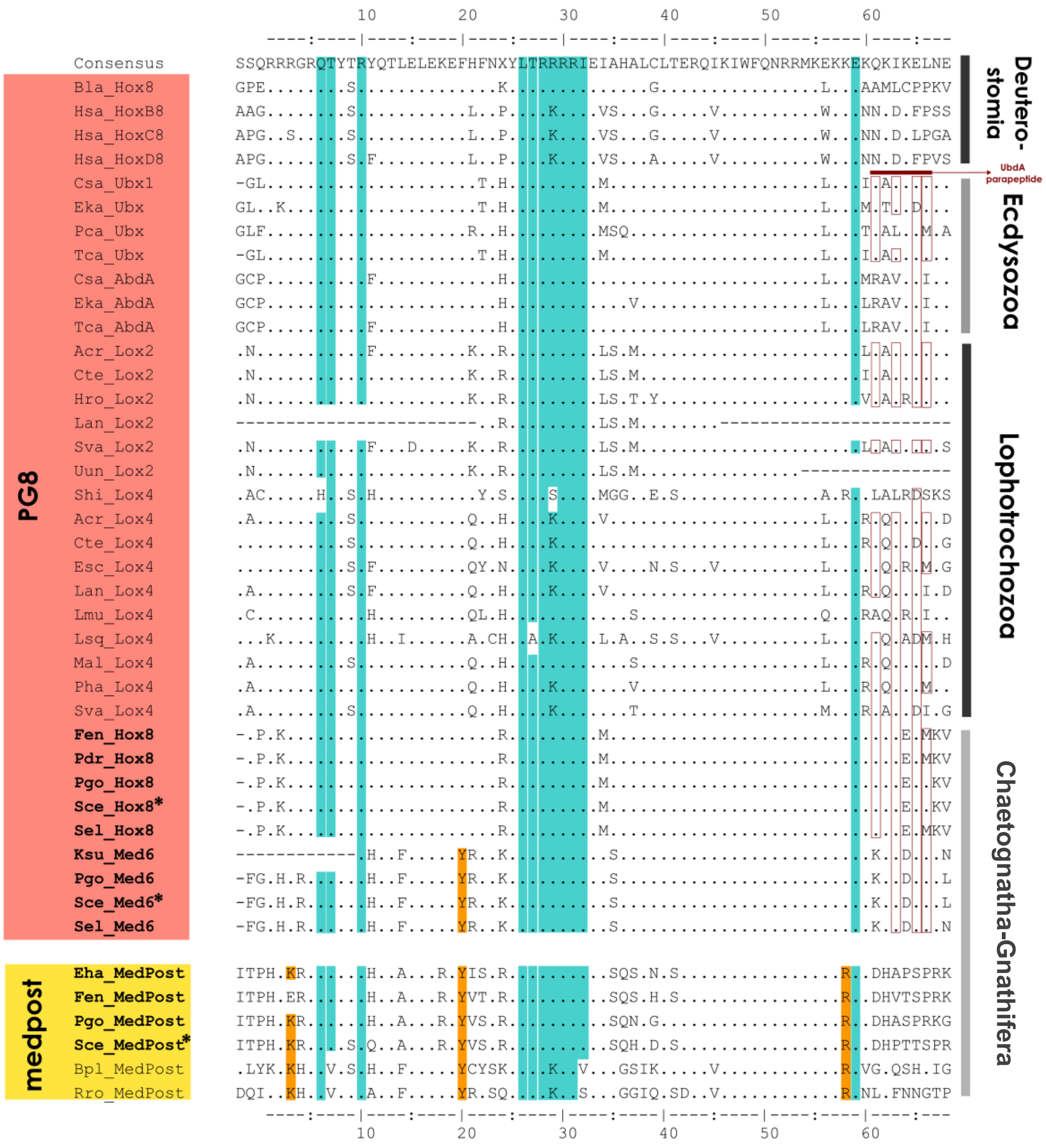

Supplementary Figure 8. Hox domain alignment of the deduced amino acid sequences of bilaterian PG8 genes and medpost genes from rotifer and chaetognath representatives. Diagnostic residues for the central class and posterior Hox are highlighted in teal and orange, respectively. The UbdA parapeptide sequence in Ecdysozoa, Lophotrochozoa, and Chaetognatha is indicated in maroon boxes. Chaetognath sequences are visualized in bold letters and *Spadella cephaloptera* genes are indicated in asterisks. Species abbreviations are in Supplementary Table 1.

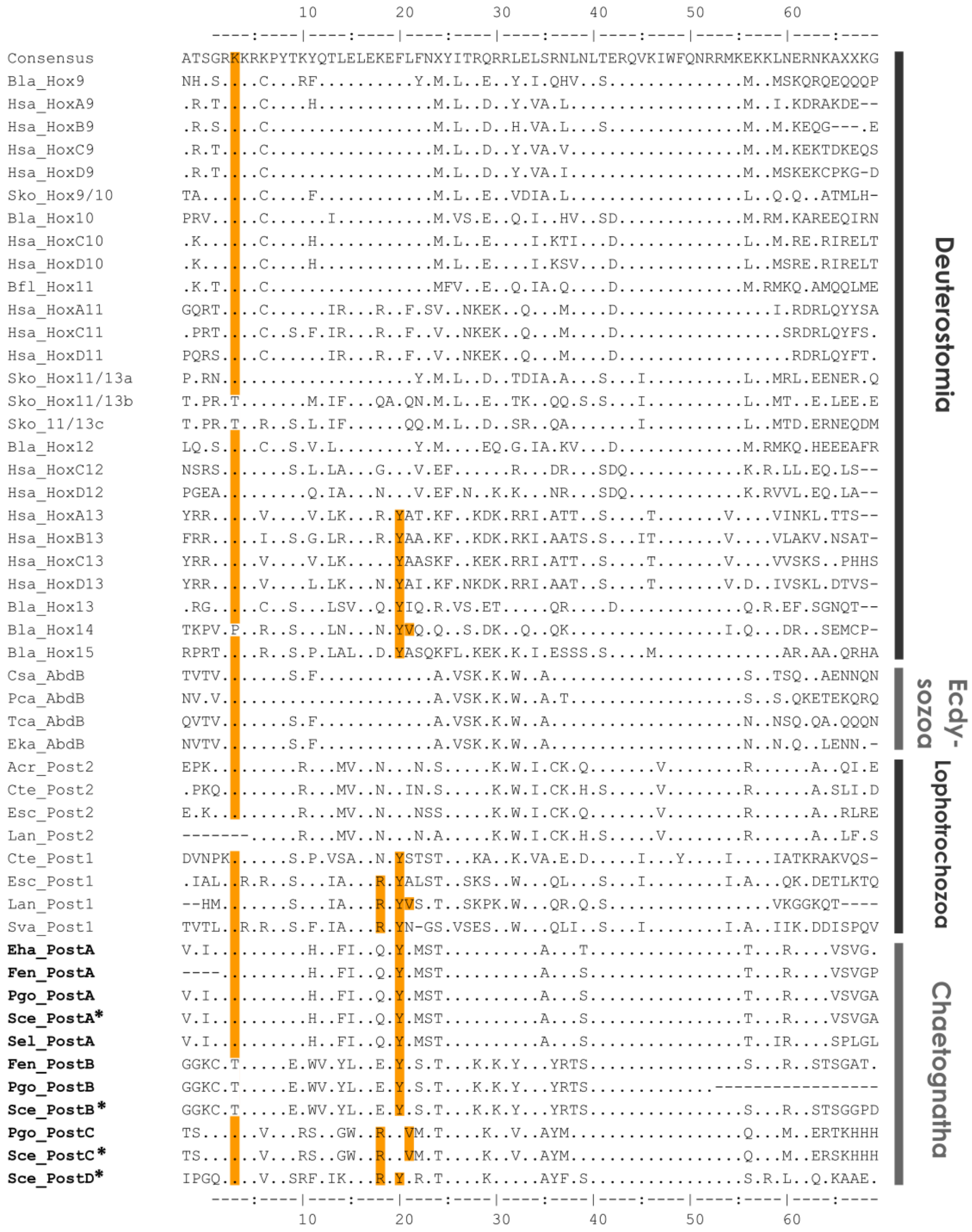

Supplementary Figure 9. Hox domain alignment of the deduced amino acid sequences of bilaterian posterior (PG9 – 15) genes**.** Diagnostic residues for the posterior class Hox are highlighted in orange. Chaetognath sequences are in **bold** letters and *Spadella cephaloptera* genes are indicated in asterisks. Species abbreviations are in Supplementary Table 1.

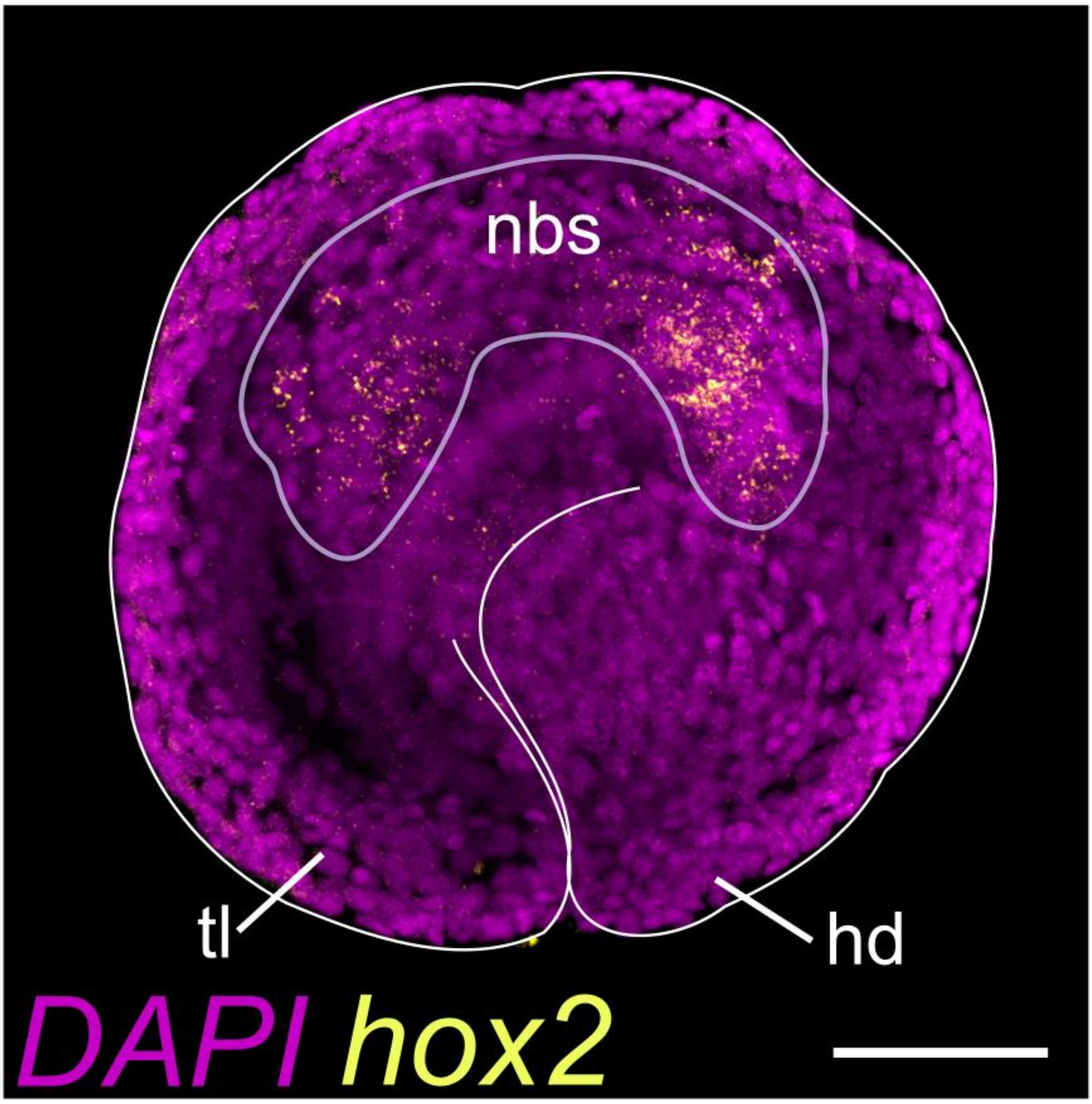

Supplementary Figure 10. Expression of *Sce-hox2* during early elongation in *Spadella cephaloptera.* Gene transcripts (yellow) are visualized with AlexaFluor647 and cell nuclei with DAPI (purple). *Sce-hox2* is expressed in sub-anterior and sub-posterior domains of the nascent VNC (*nbs*). Scale bar: 50 µm. hd, head bud; nbs, neuroblast of the developing VNC, tl, tail bud

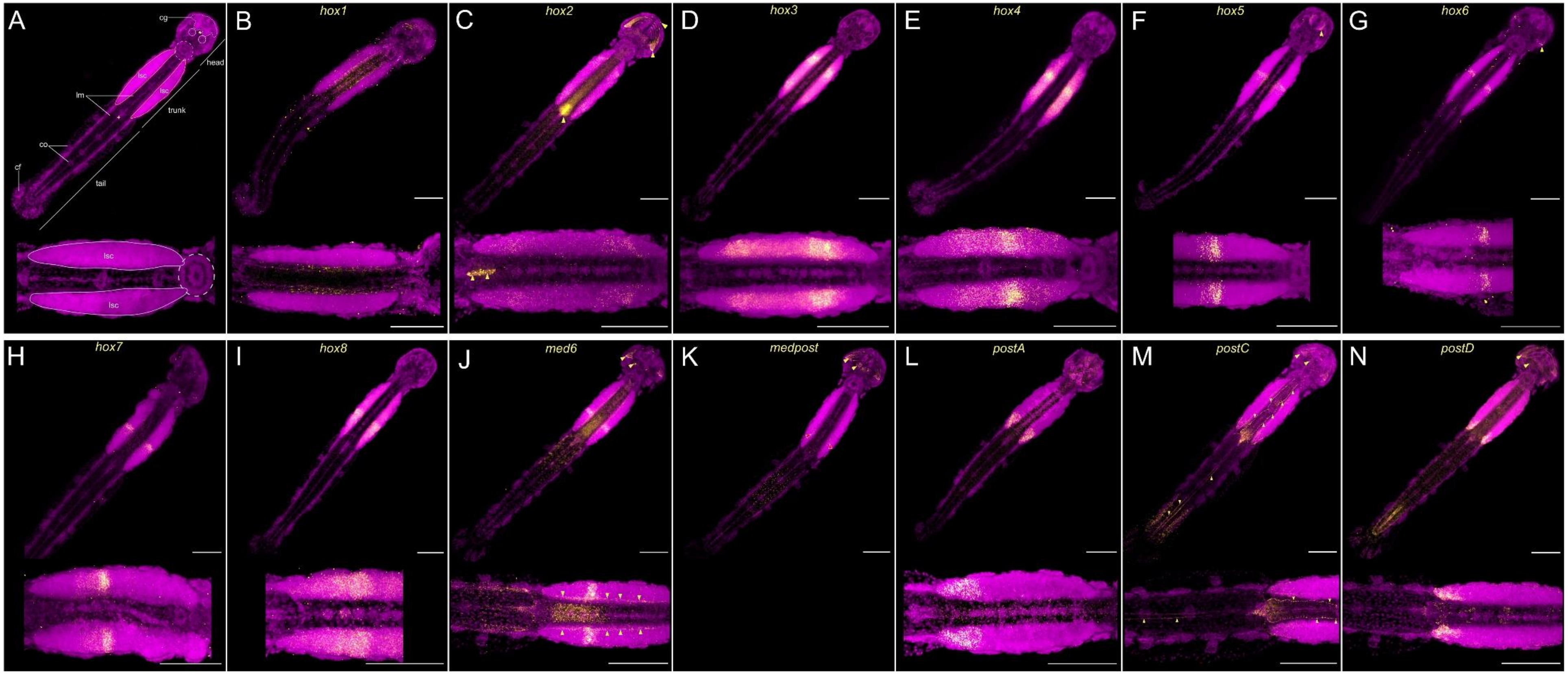

Supplementary Figure 11. Expression patterns of Hox genes in early juveniles (7-10 dph) of *Spadella cephaloptera*. Gene transcripts (yellow) are visualized with AP–Fast Blue (B, D – I, L) and AlexaFluor647 (C, J, K, M, N), and cell nuclei are counterstained with DAPI (purple). (A) General morphology of an early juvenile, showing a dorsal view of the whole animal (top) and a higher magnification of the trunk (right), both from DAPI-based maximum intensity projections. Eye locations are encircled, and the corona ciliata is outlined with dashed lines. The asterisk and crosshair mark the positions of the mouth and anal opening, respectively. (B – N) Expression of individual Hox genes (*Sce-hox1* to *Sce-postD*) in the ventral nerve center (VNC) of early juveniles. Expression domains are largely staggered along the anterior–posterior axis and show similar spatial arrangements to those observed in hatchlings (see Fig. 4). Yellow arrowheads indicate autofluorescent structures or background signal. Scale bars: 100 µm. cf, caudal fin; co, ciliary tuft/fence organs; cg, cerebral ganglion; lm, longitudinal muscle; lsc, lateral somata clusters.

Supplementary Note

Chaetognath and rotifer Hox amino acid sequences identified from the transcriptomic datasets. Sequences included in the phylogenetic analyses are indicated in asterisk:

Eukrohnia hamata

>Eha_Hox5*

VHMSHDTVNGVETKRARTSYTRYQTLELEKEFHFNRYLTRRRRIEIAHALGLTERQIKIWFQNRRMK
WKKEHKMAAVHMGFQHGMDCDPLAAAHSQLISYY

>Eha_MedPost*

AAAAYHLDPLSIYPWMAMAGLITPHRKRRQTYTRHQTAELEREYISNRYLTRRRRIEISQSLNLSERQ
IKIWFQNRRMKEKREKDHAPSPRKGHK

>Eha_PostA*

DLAHPHVSHHPPHPHVGTAALGPRSWCANGSSYGTFDVPGGHHVSPATAAAAVSGGLPLVSDHYG
ENADRLLFSFSGFHQHHAAPYGVTIGRKKRKPYTKHQTFILEQEYLMSTYITRQRRLELARNLTLTE
RQVKIWFQNRRMKTKKLRERNKVSVGGADSTADSN

Krohnitta subtilis

>Ksu_Hox4*

MDGSHESLLSEEEAATHQPGAPSLNRSALRRANGVNFAGEPKRARTAYTRHQVLELEKEFHFNRYL
TRRRRIEIAHALCLTERQIKIWFQNRRMKWKKDHKLPNTKTVKSRA

>Ksu_Med6*

RHQTFELEKEYRFNKYLTRRRRIEISHALCLTERQIKIWFQNRRMKEKKEKKKIDELNN

Paraspadella gotoi

>Pgo_Hox1*

MNSLSTTNVDYSYAAGVGVGPGNYFHPHHHHSHLHHPHHHHHHHQPLEAFQAEQSAYSHHHYYS
THPHHHPAAPPAYQPHPASYSTENFYAHHHHLQYGQTTPGTTTTPAEATPPIDSGPNPSSSPFGGTLSP
SPSPSAPFNVSVPSTTSSAPQSIDSSALQASSAKGSPGVGFPLDRLTAADASSSGAGFSYTGSIRMTMR
GDAGGAVGSYPGAAGSFYGALQSPPVSSAPSSSPPLNGYTTAPHLVQHAGGADSPLGAAAAAAAE
FEAAPSSASLFLCDTESSSTPRRELECSATNGVVSAASAAAVAASMHPTLPPTPPNYHHPAHHPVHH
HLAATTVNGASVAFSTPMAPTDQQPALLESPLHPHHHHHHHQLQHPYHHSQQQQHHLLNGMAS
MPAEATAAEAEKPRAVPNYKWMQLKRSTVKSGSNVNNSFAYNSNNNINPTPACPSSLPGHGAHGPA
HGNNANLHNSAGNNNSNSLGRTNFTNKQLTELEKEFHFNKYLTRARRIEIATTLGLNETQVKIWFQN
RRMKQKKRQKEAKFNSENAEILAAAAAANGSSGGSSVAAAAAAAGTTDALSGDLVCGVAAGSTSS
SSSPINSPSELGVGVPDIAGVESTDSGGQAGRVSPVGGAVKREGALDETGTP

>Pgo_Hox3*

MGPHGAHLSMPHPAEQQQPPNGAHPHHGHHPHHMGGGGGGPQANGHHGAVHHPMQVLNPNHA
LGSQSHHRMAAEAPDSVLHPHHSAAAAAAAAAQLSAAHTAGGVVHGGGPGVSPPHHGSPMYPKE
IYPWMRESRQNSKQKMAEFEQPTKRARTAYTSAQLVELEKEFHFNRYLCRPRRIEMAALLNLSERQI
KIWFQNRRMKYKKEQKLKGIDVKDGEEGEGDEPDTSSSPGVVTSCSGGHHAGHGHGCGAGSGVR
EINDDDADKASDAGSIGPGSGGPPSHTSLLSRGGVGGPDLPATSTSGDAMRGDAMDAMDANAAGP
TSSSSSAQSPSPPPSLTATNPLKVNGQGPASSASSAGSPAAGGGGGRLRGLSGLVHGMDVGVGGAGG
GGATSGGQPKSSPGASPIRNQQIVNARQHQQQQLQQQFAAAAAAAAAANGGNPPLSPFSTPPPAG
HAYPHPQRLTPSGVGGNRPSSPTPASTCVTQGSNPSSHMSLINGSPQMQTPQDALTAPNQRSMIATSG
YSAPMTHMSSPQTISSPSSSSSHGLLHHHQLESCSPPPLLVSIPPLVNVGGADPSVAYPHHLQQHHQQ
QQQQQQHQHQQLHHQEMQQHQHHHHAMQHQQQQQQQHRNQQSPIFDKISCFDVFPQNNNAGL
NSPSPPTIGVSYAHMNLGGGVGGGGGGGAGGLMNNPHMSGPPHHHHPHHGNSPMSRGPGHGPPPS
SSSSPSPSVSSAAPPPPPAANQHLPNSYCSMAQGGNGVGAPAAVGVAAGAGYNGGLSTPPKLTHL

>Pgo_Hox4*

MSAFLMDSSSAAASPYNTLLDTKFPPAALDDYGVSGYGHHPHHHPGDYYHHHHPHHHHHSRASH
HHYNPYDQRGYEEHYGHHNASNYACSVPGVPSHHNMSPLHQSQQQQQQQQQQTSQHCSNGPAN
GMSNGGGGGAPVGGAGGDPGPLNQDPSPVTPQAPVSMNSGPGGGVAGLPPHHHLAPGANMLIGA
TADSQSHHQTSSTTGLSLSPGLCGMEPAGGGLPMPPHSLAASNGMPVASLSQSLQQPTSVAAGHHQ
QQQPQPHQQHHSQQPPPPPNQPPPPLVLDPHPSPPLHPHDHPHHPHHDPRLGHGFDPDDPDDLEMA
DHNGNGQAPVIYPWMKKVHVATSNGTNFAGEPKRARTAYTRHQVLELEKEFHFNRYLTRRRRIEIA
HALCLTERQIKIWFQNRRMKWKKDHKLPNTKTVKSRAAVVAQQAEQAAAAAAKAQAANENNNS
EQQQQQQQQQQPQQTHLQNMNSAGNSVQNIPPDISAPGSFSMDNDIDSLEDLQ

>Pgo_Hox5*

MSSYFVNSLSAAAATNQCYGGQQQQQQQPGGMDPAGQQQQQHAFSSQHDPNSIAAAFQSASTAA
HQLPGAYAAAAAAAHHGGFGMAAYHQQQQQHHQQLSVQQQQQQQHNGVDNGGGFVEPPLSPSP
SMESGSPSPASMGGFGADLVANTVANAGAYSKVPIPSVSSIAGHQQHNHLHQSHHLHHSSASRHQQ
QNAASPQALHNSHHMAGLGAATPQPPPAHHHHHSTHLAPPPSPSTAGGGGVSPGGQGGVGGGRGS
EHFHGGAGVSPTTQQTTPSLGANHQHHHHLPQDQKPAPVARQDNNNGASSSSSSSSSSSTSSSSSS
NSEANPALTTTSNNNNNNSSSSSSNNSSSSSASSSSNSSSNASSSSGSSSSNSNGAKGSSSSTSGGGGG
GRDAGGSSPASTGSSDGDGETTPTTTTSSTPQVQIYPWMRKVHMSHDTVNGVETKRARTSYTRYQT
LELEKEFHFNRYLTRRRRIEIAHALGLTERQIKIWFQNRRMKWKKEHRMAAVHMGFQHGMDCDPL
AAAHSQLMWTMQSGW

>Pgo_Hox6*

MTSHYSHVFSQNFSHGQESYFGGYDAGGGIGLGGVGSGVGKFADIPPSGAGFLSTSPGDHDSPSAA
AAAAAVAAAAGDNAFSSCYPGATTTAANSLDVHPHHSQAQQQLHHQHHQRSLSDGGGGGAPGDA
SPGLATPTSSAVSHPYLGHDVIGPYSPTSSSRPSSSTPYNAFSVSAHHAAAALHSQTAPLASTGNSIAS
LTPPMDRCYDMAASMATPKTPLDAAASAGASNFYHMSCAYQSIGAAEFDVYGGAAASAAALAPK
ASDQNSHPGLQQHQHPQQHHHHHQQSLSPDSRSLALPRSPPLSHHANLHAAANAAVAAALDDPFN
GVRKFKTFADNGAVASEHHLQSCGSPPSEAYTSGGESSPCPSLPRSPGPMGLESQQGQHPHHQQHLP
ANVSGNSHNHHQHNHHQHHHQSSPVPSHLNPSTLKSEEATSPLLGRSIGSSASPTTTTTTSSSASSRL
SADASVKGAASKASEADRPSAASSSGPTSASASSSSAPSSSSSSSSSSSASSSSSNRGSDPSPSAATAAA
SAAAAATPPGSSSPSQEKKGEEEEGEEDGEKDEDSPAIFPWMRGNTGDLGIDQKRTRQTYTRHQTLE
LEKEFHFNRYLTRRRRIEIAHALGLTERQIKIWFQNRRMKWKKENNLKSINDAKPEFKEGNFLNCH
WRPGGMGPMFGPPPNHPAALNHPFANNFPNHHYHQFQQQFFGGQPTQCAPY

>Pgo_Hox7*

MNSFYSPSAAANYMSGLNGGFSSGTNAMHPDVMSPAGAASTSTSGAYDPMRTLSSYGAPVSPASD
LGASTAETHGAALTPHFPRFSTYDRGGGGDSDGSPSPHATPTSSQSLLLGAGDAGGGGVVVPVSSSS
PSSSSTSSSSSSSSSSQQPQPAHQHRNSLPHHLHHHATAASNLCSPGALGHQQHHHHHHHHPAFFPEG
VVSGNGALHRGSPGSGSSPTSSPSTSLHHNNNNNNSSSSNSGGVFPSPVVDDALRVGCPVTSSSNAGV
HSSSVHSGGGGASSSTVHHHHFGGAACHPGDTPPHNGVPSSSSSSSPSSSSHNHLNGHQQQQQQQ
PDNSQHPLNLLQQQQQQQASHLEQQHHGNGVVNGPASSYPSHPLTPQPPPHMGAPGVNGVAGPHG
VMGAGHPGHGPQEGQAAPVDHSLNSQTPIYPWMRSQFDRKRGRQTYTRYQTLELEKEFHFNRYLT
RRRRIEIAHALCLTERQIKIWFQNRRMKWKKEQKAALGVGMPGPGHLVMGGMPPGLMAHAGHPG
MHHGLTEMELCKKEVMGE

>Pgo_Hox8*

MTTYNVETILSRGGDTPVLDNGYPTSLNHHSSNAYGPCGYPQPTPHSQPLHQNQPLSSQQQHPQQS
TPPHQSHTPGLPADVVNGGGGGAGGGAGHKYPTTNGWGNAGPQQHHPQQQQQQPQHHPAMNQN
SAPSSYSCHGAALSASAAAVASSPRCPSVGSMGGAAAHGADYSAYQPPGATGAPGTPNENDYGKM
TLCGDTYGGAIPGHIGQAAGHPYSHHQQQQQQQQQPTVQGHPNRWSPHPNQTPPHGGQHSPVLGN
SYSSHQNQGLGAVGLSHDGFQQGHPVSQQQDPNGHSPVHNISSQCNYNGHQQSQGPPTPFYPWMS
LAGVNSPRKRGRQTYTRYQTLELEKEFHFNRYLTRRRRIEMAHALCLTERQIKIWFQNRRMKEKKE
KQKIEELKVEGGGGSGGGGGGKDAMHKLDSSPGSADGVCCEG

>Pgo_Med6*

MAASYGLNHFLPNYQSEPRLAESMHSLTSQYHDFNSAWSKSASTAADAAAAAAADNFTESVKSSL
FNNNFAASLGRAAAANSGAGAGGAHHAHHHHHGSHSAGGGVPYSSYSSLASFPSSAAAAAYGQA
NANPHHPSAAAAAAFGGQGLLDSAAALQHQALQHQAAAASLSQCSFSDISNMTCHTATIYPWMAS
AGAEFGRHRRRQTYTRHQTFELEKEYRFNKYLTRRRRIEISHALCLTERQIKIWFQNRRMKEKKEKK
KIDELNLEDKEKQEKLGGGSGKAASSSSASVDKDAASDDASEEEDVDDPLLENAAEILKDVKKAA
LAAQQQQQQQVHQQQHHSQHQQQQQHHHHHQQHQQLLATPTQS

>Pgo_MedPost*

METTLYNNHPHPHHHHHHPHHHPAHHQYYGSPDVHSMSTTPTTGSNFGLNESLSPFGGGGGAALG
HPPTPPGSSGYCHHPASSMGGSGGSPGLSGGGVASALVNGPMGGAATPISLTADYHHHPHFHGVVG
GGGGALDHQRLSGSGASKSSYGAMNFNAHHPHHPYQIDAAAPVPHPAAPLLHAAKSHAASAASFD
SYAASVDERQSELMNHQASSLQHHLQQQQQHQSQNGSSHDHAVHGGNSHPHGQHHHHHHHQQQ
QQSEGGVNSQSPPPSLPNVAPSSSAHLPHPHPHSSHSPVSPLPAISPPTVPGSVSPPQQQQQQQQHHTP
SAPPTGCLSVMSSRYSAPSSSGAPSCSQSASSLTSSSLDVACSSAVVAPTNSRASASAAATAAALSGG
VAAHGNNNVVGAASSAAADCGVGSAIAAASNGTSAALTMAAAAAAAAAYNNYDPVTMGWMAM
AGLITPHRKRRQTYTRHQTAELEREYVSNRYLTRRRRIEISQNLGLTERQIKIWFQNRRMKEKREKD
HASPRKGAGK

>Pgo_PostA*

MEGLVAKYPDPIHLPLSAAAPSNLPTAPAAATAAVAASLPTAAAPPSASPVQTTSLPSPVPHPISSLPGQ
SASSAAAAAAAAAAAATRTSVAAAASYYTKYETAVKNEYIKNEFMKSDYLPNSISAAAAAGVAAAK
DSTSTHPHYADQFSLWKTSAADSTSSMAASSMYTANLPALGPYSSLVDRAAAAASANPFPSAAAAY
SAAASSYYTPHHHHHQAAAAAAAAAPTHPHHHHPPHPHVGAAALNPRSWCGANASFNGTFDVPG
GHHVSPATAAAAVSGGLPLVGDHYGDNADRLLFSFSGFPHHHAAPYGVTIGRKKRKPYTKHQTFIL
EQEYLMSTYITRQRRLELARNLSLTERQVKIWFQNRRMKTKKLRERNKVSVGADSTADSN

>Pgo_PostB*

AAAAAAAAAAGLTSTQQYLAGVTGYSSHRGLDFNKIDTNCLDPTNFFHACTSGGKCRTKRKPYEK
WVTYLLEEEYLSNTYITKQKRYELSYRTSLTERQVKIWFQNR

>Pgo_PostC*

MYSVSSAPASCGFPSNAAAHLGLHNPHHQLSTGPHHHAHPAQTDHSPLYHHHHPFSSLSAAAAAAG
GHAHGLGGLGGVYSTHPFFNASAASRSGGDVSGGGGVLGGHAHPSTTASAASAIPAPHPLTAPTPS
WNGSSPSGDLGLETGLLQPGQHHPDPRSDNHHHHPSPPLQHHQHPSQQQQQHSKGPLMGASTPTL
PHQQHPVDPAATTAASAADSAFPCSPLPGAEYSAAWTQFHAGSSVGATVGANEVGYGTVKPTSVN
AFGQQPRQQQQQHSFESAANHRRSPVDASLPLRSTNANVSGVNADGGANNNNNNNNSSNVRLNL
SPSDCGVDDAIVDDRLSPDDDDDDDDRCHSALGELTPTHPNLNHSSTTPTQLNHQSDVTPPAHASA
HPVMDDFYGAGPYGPYGVGAVAAAAVAAAASTSSASTTTSSSTSSALTSYMASTANAWTTPSSIGA
NPYGLSVSSGGFGAAGLGAGVGGVGGANFYSQSNSSLGSTASLGSYFPSPLADGLAKPSPLTSGGV
TTAILPPHIPGLTHPFPHHHPHDPHNHGPAVIELTSSGRKKRVPYTRSQTGWLEREFVMNTYITRKRR
VELAYMLNLTERQVKIWFQNRRMKQKKLMERERTKHHHHHHGAGQEIKTM

Pterosagitta draco

>Pdr_Hox1

SNNNPNNGNNLGRTNFTNKQLTELEKEFHFNKYLTRARRIEIATTLGLNETQVKIWFQNRRMKQKK
RQKEAKFNSENADILAAANSANSA

>Pdr_Hox3

RESRQNSKQKMAEFEQPTKRARTAYTSAQLVELEKEFHFNRYLCRPRRIEMAALLNLSERQIKIWFQ
NRRMRYK

>Pdr_Hox4*

LSLSPGLCGGNMDSSNQQQQPQHPPHSMGSNGLPVSSLSQSLQQPTSVASHQQHQVQQQQQQPHH
SQTPNGPPALVLDPHPSPPLHPHDHPHHPHHDPRLGHGGYDPEDSDDLDLADHNGNGQAPVIYPW
MKKVHVATSNGTNFAGEPKRARTAYTRHQVLELEKEFHFNRYLTRRRRIEIAHALCLTERQIKIWFQ
NRRMKWKKDHGLPNTKT

>Pdr_Hox5.1 (Note: The sequence represents distinct non-overlapping regions of the same gene, not paralogous copies)

DPAANASPSSSGGSGSSGSSSNEENDGGESPTPAAASAPQVQIYPWMRKVHMSHDTVNGVETKRAR
TSYTRYQTLELEKEF

>Pdr_Hox5.2 (Note: The sequence represents distinct non-overlapping regions of the same gene, not paralogous copies)

RSEIAHALGLTERQIKIWFQNRRMKWKKEHKMAAVHMGFQHGMDCDPLAAAHSQLISYY

>Pdr_Hox6.1 (Note: The sequence represents distinct non-overlapping regions of the same gene, not paralogous copies)

PSSAQDKKAEEEDGDEDGEKDEDGATIFPWMRGTTGDLGIDQKRTRQTYTRHQTLELEKEFHFNRY
LTRRRRIEIAHAL

>Pdr_Hox6.2 (Note: The sequence represents distinct non-overlapping regions of the same gene, not paralogous copies)

KIWFQNRRMKWKKENNLKSINDAKPEFKEGNFLNCHWRPGGMGPMFGPPPNHPAALNHPFANNF
PNHHYHQFQQQFFGGQPAPQCAPY

>Pdr_Hox8*

FSSQGHPAISSQHQDLPNNHSPIHSISSSQCNYNGGHQQSGGPPTPFYPWMSLAGVNSPRKRGRQTY
TRYQTLELEKEFHFNRYLTRRRRIEMAHALCLTERQIKIWFQNRRMKEKKEKQKIEEMKVDGVSPN
KDSMSKLDNS

>Pdr_MedPost

LEREYVSNRYLTRRRRIEISQSLHLSERQIKIWFQNRRMKEKREKDHVTSPRKNK

>Pdr_PostA

LFLRADRLLFSFSGFHQHHGAPYGVTIGRKKRKPYTKHQTFILEQEYLMSTYITRQRRRRRRGRRRR
RRKSPATHLSS

>Pdr_PostC

FSNPLSDGGVTTAILPPHIPGLTHPFPHHHHPHEHGPAVIELTSSGRKKRVPCTRSQTGWLE

Sagitta elegans

>Sel_Hox1*

MNSISTSANVEYASYPTGGVLAPGQYFPPTSLDAFQMEQNSFSSHPYYHPAHPHHYHSPHHPPSHQH
PHHHHHPPPGYSPQHPSYAADASASHSLSSFYQQQQHQQMVAATAGISPSSSSSILSPQQQQQQ
QLAQQQLLATSGVSAESAPTNPSSSSPFGGTLSPSPSPSSTSFNHLVASSSSASLNHTVPSSGASSGITG
TSFGGISSGVSTTASPASTASSIKQMTAHQYHENPYHHRISPSAVSTDSPSLQASASSSATVAAAAALG
ISYSGSIRMTPAFRGGGGDGVVGGVVGGIGGVYTPSGFYGSHQPELASASGIAPSASPPTNAFNASSS
SLVGRNNSTSNSPTHPSSASSSNAHFDAASTTFSSYRQQQQHCDTDPTSMGVPDLEEGSRGAGGSPL
TASSSAKASSLHPTLPPTPPNYHPHSNHPLAHLAHASSVNGLSVTPHFALDRCQQQQQPQQPHHLHQ
QQQQQQQQQSAILESPLHSHHPSHLFMHQAPHAHLLHHHQGQLPAEYGSGAPEAEKPRQVPNYKW
MQLKRSSSKSAGTTNNNNNAGSAAPYAYNGSSLPNIVSNPNVSNGALNNGSGSNNNNNSAAAPNN
NHNSNPNGNNNGTSTANNNNLGRTNFTNKQLTELEKEFHFNKYLTRARRIEIATTLGLNETQVKIWF
QNRRMKQKKRQKEAKFNSENADILAAANSANSASSSSSASAGVSDVANAPSPTDESGAGNAENEA
RSPEGSAGSVEKAVSSPSSRLNRATPDGKPKGEIE

>Sel_Hox4*

MSAFLMDSAAMGASPYNTLLDTKFPPTPIDDYGVSGYGHHPHHHPSDYYHHHHSRAAHHHYNPY
DHNRGYGGHSDEHYSQQHHNPAASNYGCSVPGIPTSHHNVSPAHQSQHCGGAAGGGGGGGAGSG
ANHINGGDPAAINSGAHSDHPSSITPQAPVAMNSGPGGVTGLPAHHHMTPGGNIMIGATADSQNHQ
QTGLSLSPGLCGGNIDSSNQQQQPQHPPHSMGSNGLPVSSLSQSLQQPTSVASHQQHQVQQQQQQP
HHSQTPNGPPALVLDPHPSPPLHPHEHPHHPHHDPRLGHGGYDPDDSDDLDLADHNGNGQAPVIYP
WMKKVHVATSNGTNFAGEPKRARTAYTRHQVLELEKEFHFNRYLTRRRRIEIAHALCLTERQIKIWF
QNRRMKWKKDHKLPNTKTVKSRAAVVAQQAENAAAAAAAAKNQVSSDNNNSAADPTSQQQQQ
QQQQQQQQQQQQTHLSNNSVPNIPPDLSSAGSFEMDNDMDSLEDLQ

>Sel_Hox5*

SEQFSGSGSSGGGGSVTPTAPSSSNHLNHHLQQQHQKSSSSRSDGISGSGGGGGGVSGSSGGGTAHS
SSSSSSAVNGASSSSSSSSSLNTSGNSASSSNSNNNNNAKATASSSSSSAPSSSTTTPSTSAGGGSETAD

SAANASPSSSGGSGSSGSSSNEEGDGGESPTPAAASTPAVQIYPWMRKVHMSHDSVNGVETKRART
SYTRYQTLELEKEFHFNRYLTRRRRIEIAHALGLTERQIKIWFQNRRMKWKKEHKMAAVHMGFQH
GMDCDPLAAAHSQLISYY

>Sel_Hox6*

SFQVDNPSAAGGLQQQQLRGCSSPPSESYTSGGDSSPCPSLPRSPAPLNGAHSLDQQQLVATPNLHH
QHQHLPHHHQNLQQHHQSHLQQQQHNQHHYPQQSSPSPLPSTLLNTPGKEDNGGRTNLFTSSTSS
TTTTASTSSSSSTSLSTSSLGVSKTTTTPTKSGDGRNGANSPNTTPTSSTSSSSSSSSSTSSKPSPSSSTSS
SSAAGATTGASTTPPGSASPSQEKKGEEDEGDEDGDKDDDGATIFPWMRGSSGDLGIDQKRTRQTY
TRHQTLELEKEFHFNRYLTRRRRIEIAHALGLTERQIKIWFQNRRMKWKKENNLKSINDAKPEFKEG
NFLNCHWRPGGMGPMFGPPPNHPAALNHPFANNFPNHHYHQFQQQFFGGQPAPQCAPY

>Sel_Hox8*

CNYNGGHQQSAGPPTPFYPWMSLAGVNSPRKRGRQTYTRYQTLELEKEFHFNRYLTRRRRIEMAH
ALCLTERQIKIWFQNRRMKEKKEKQKIEEMKVDGASPNKDS

>Sel_Med6*

ANAHNHHHSASAAAAAVGFGGQGLLDSAAALQHQALQHQAAAASLSQCSFPDISNMACHSAPIYP
WMATAGAEFGRHRRRQTYTRHQTFELEKEYRFNKYLTRRRRIEISHALCLTERQIKIWFQNRRMKE
KKEKKKIDELNNEDKEKHGSGSGSGSGSGSGDKSISGKASSSSSSG

>Sel_PostA*

HYGDNADRLLFSFSGFHNHHAAPYGVTIGRKKRKPYTKHQTFILEQEYLMSTYITRQRRLELARNL
SLTERQVKIWFQNRRMKTKKIRERNKSPLGLTQPPKIQIDHEPLCLIDISRHLR

>Sel_PostC

MNSAAAAASNPYGLSTSAAAAAAAGGYGAAAASGFYSQANSSLGSSSTLGSYFSNPLTDGLTKPSP
LTSGGVTTAILPPHIPGLTHPFPHHHHPHEH

***Rotaria rotatoria* (Rotifera)**

>Rro_MedPost*

MDGTTSAVNYLLSPTPNDYYDHHSLQPIQPLGNLHYYQSDPIDCFYDHQKADFQSTSAYIYHPTQQP
PFGYGSSIYDMQSSQQFVQESNILLLQNQPRLPPPVYDLPSSSHNPQSTSSQPKVLKREIDETASTTER
NHVYEWMKGDQIRRKHRQVYTRAQTFELEKEYRFSQYLTRKRRSEIAGGIQLSDRQVKIWFQNRR
MKEKRENLKFNNGTPSIPKQRNDYTS

Supplementary References

1. Saadi, A.J., De Oliveira, A.L., Kocot, K.M., Schwaha, T., 2023. Genomic and transcriptomic survey of bryozoan Hox and ParaHox genes with emphasis on phylactolaemate bryozoans. BMC Genomics 24, 711. https://doi.org/10.1186/s12864-023-09826-z

2. Currie, K.W., Brown, D.D.R., Zhu, S., Xu, C., Voisin, V., Bader, G.D., Pearson, B.J., 2016. HOX gene complement and expression in the planarian Schmidtea mediterranea. EvoDevo 7, 7. https://doi.org/10.1186/s13227-016-0044-8
