# Supplementary material for: Anterior-posterior patterning in the chaetognath Spadella cephaloptera informs bilaterian nervous system and tail evolution
Source: Commun Biol. 2025 Dec 26;9:122. doi: 10.1038/s42003-025-09398-6 (PMC12852818; doi:10.1038/s42003-025-09398-6)
Supplement: Supplementary file 2 — Description of Additional Supplementary Files [file 42003_2025_9398_MOESM2_ESM.docx]

**Description of Additional Supplementary Files**
File name: Supplementary Data 1
Description: HCR probe sets and their corresponding amplifier hairpin types used for the HCR RNA-FISH experiments in Spadella cephaloptera
