# Supplementary material for: Anterior-posterior patterning in the chaetognath Spadella cephaloptera informs bilaterian nervous system and tail evolution
Source: Commun Biol. 2025 Dec 26;9:122. doi: 10.1038/s42003-025-09398-6 (PMC12852818; doi:10.1038/s42003-025-09398-6)
Supplement: Supplementary file 4 — Reporting Summary [file 42003_2025_9398_MOESM4_ESM.pdf]

nature portfolio

Corresponding author(s): Tim Wollesen, June F. Ordoñez

Last updated by author(s): Nov 25, 2025

Reporting Summary

Nature Portfolio wishes to improve the reproducibility of the work that we publish. This form provides structure for consistency and transparency in reporting. For further information on Nature Portfolio policies, see our Editorial Policies and the Editorial Policy Checklist.

Statistics

For all statistical analyses, confirm that the following items are present in the figure legend, table legend, main text, or Methods section.

n/a	Confirmed	
☒	☐	The exact sample size (*n*) for each experimental group/condition, given as a discrete number and unit of measurement
☒	☐	A statement on whether measurements were taken from distinct samples or whether the same sample was measured repeatedly
☒	☐	The statistical test(s) used AND whether they are one- or two-sided<br>*Only common tests should be described solely by name; describe more complex techniques in the Methods section.*
☒	☐	A description of all covariates tested
☒	☐	A description of any assumptions or corrections, such as tests of normality and adjustment for multiple comparisons
☒	☐	A full description of the statistical parameters including central tendency (e.g. means) or other basic estimates (e.g. regression coefficient) AND variation (e.g. standard deviation) or associated estimates of uncertainty (e.g. confidence intervals)
☒	☐	For null hypothesis testing, the test statistic (e.g. *F*, *t*, *r*) with confidence intervals, effect sizes, degrees of freedom and *P* value noted<br>*Give P values as exact values whenever suitable.*
☐	☒	For Bayesian analysis, information on the choice of priors and Markov chain Monte Carlo settings
☒	☐	For hierarchical and complex designs, identification of the appropriate level for tests and full reporting of outcomes
☒	☐	Estimates of effect sizes (e.g. Cohen's *d*, Pearson's *r*), indicating how they were calculated

Our web collection on statistics for biologists contains articles on many of the points above.

Software and code

Policy information about availability of computer code

Data collection	For HCR probe generation, a custom code was used (https://github.com/rwnull/insitu_probe_generator).
Data analysis	For phylogenetic analyses, Blast v2.16.0 (https://blast.ncbi.nlm.nih.gov/doc/blast-help/downloadblastdata.html) was used to search for genes of interest (anterior-patterning and Hox genes) in the Spadella cephaloptera transcriptome. For identifying Hox genes from several chaetognath representatives, Trimmomatic v0.39 (http://www.usadellab.org/cms/?page=trimmomatic) was used for adapter removal, quality filtering, and trimming; rnaSPAdes v4.1 (https://ablab.github.io/spades/) was used to perform transcriptome assembly; and DIAMOND v2.1.11 (https://ablab.github.io/spades/) was used to screen for Hox genes in each species. MAFFT v7.490 was used for multiple amino acid sequences alignment, which is integrated in Geneious Prime v2023.0.1 (https://www.geneious.com). ClipKit v2.2.3 (https://jlsteenwyk.com/ClipKIT/) was used to trim the multiple sequences alignment. IQTREE v2.3.6 (https://iqtree.github.io) was used to perform Maximumum-Likelihood-based phylogenetic trees. MrBayes v3.2.7a (https://nbisweden.github.io/MrBayes/index.html) was used to perform a Bayesian-based phylogenetic tree of Hox genes.<br><br>For image processing, Fiji (ImageJ) v1.54f (https://imagej.net/software/fiji/) was used. For generating figures, Inkscape v1.4.1 (https://inkscape.org) was used.

For manuscripts utilizing custom algorithms or software that are central to the research but not yet described in published literature, software must be made available to editors and reviewers. We strongly encourage code deposition in a community repository (e.g. GitHub). See the Nature Portfolio guidelines for submitting code & software for further information.

Data

Policy information about availability of data

All manuscripts must include a data availability statement. This statement should provide the following information, where applicable:

- Accession codes, unique identifiers, or web links for publicly available datasets
- A description of any restrictions on data availability
- For clinical datasets or third party data, please ensure that the statement adheres to our policy

The assembled transcriptome for Spadella cephaloptera is accessible on Zenodo (https://zenodo.org/record/7602960#.Y90U0oSZOUk/DOI:10.5281/zenodo.7602960). Newly obtained S. cephaloptera sequences used in the phylogenetic analyses have been deposited to GenBank (PX219847-PX219863).

Research involving human participants, their data, or biological material

Policy information about studies with human participants or human data. See also policy information about sex, gender (identity/presentation), and sexual orientation and race, ethnicity and racism.

Reporting on sex and gender	This study did not involve human participants, human data, or human tissue.
Reporting on race, ethnicity, or other socially relevant groupings	This study did not involve human participants, human data, or human tissue.
Population characteristics	This study did not involve human participants, human data, or human tissue.
Recruitment	This study did not involve human participants, human data, or human tissue.
Ethics oversight	No ethics oversight was required for this study, as it involved only non-endangered marine invertebrates (Spadella cephaloptera) and did not include human participants or vertebrate animals.

Note that full information on the approval of the study protocol must also be provided in the manuscript.

Field-specific reporting

Please select the one below that is the best fit for your research. If you are not sure, read the appropriate sections before making your selection.

☐ Life sciences ☐ Behavioural & social sciences ☒ Ecological, evolutionary & environmental sciences

For a reference copy of the document with all sections, see nature.com/documents/nr-reporting-summary-flat.pdf

Ecological, evolutionary & environmental sciences study design

All studies must disclose on these points even when the disclosure is negative.

Study description	We analyzed gene homologs involved in anterior-posterior (AP) patterning in S. cephaloptera using sequence data from the transcriptome. Phylogenetic analysis was performed to determine gene orthology, incorporating sequences from various bilaterians. For animal collection, adult specimens were collected from the intertidal zone in France and kept in controlled conditions for breeding. Embryos and post-hatch specimens were collected at different developmental stages for gene expression analysis. To study gene expression, RNA was extracted from the specimens, and riboprobes were synthesized for use in fluorescent in situ hybridization (FISH). Both RNA-FISH and HCR-FISH techniques were employed to examine gene expression patterns in the embryos and post-hatch stages. Specimens were imaged using confocal microscopy, and images were processed and analyzed for visual presentation.
Research sample	The research sample consists of adults, early stages of post-hatching, and embryonic specimens of Spadella cephaloptera. The sexually mature adults collected from the wild were used to produce embryos and early post-hatching stages, which were used examine gene expression patterns during the early development of the organism. Public RNA-seq datasets (Marletaz et al. 2019; DOI: 10.1016/j.cub.2018.11.042) from selected chaetognath species were incorporated to support the phylogenetic analysis of Hox genes. These datasets were sourced from the SRA (Sequence Read Archive) and provided transcriptomic data to enhance gene orthology determination.
Sampling strategy	No formal sample size calculation was performed. Sample sizes were based on standard practices in qualitative and descriptive developmental gene expression studies, with multiple specimens examined per stage to ensure reproducibility of observed patterns.
Data collection	The identification of genes of interest were performed by TW and JFO. Probes were designed and synthesized by JFO and TW. Gene expression data were obtained through whole-mount RNA Fluorescence in situ Hybridization (RNA-FISH) and Hybridization Chain Reaction (HCR-FISH) conducted on embryos, hatchlings, and early juveniles by JFO. The data were documented and recorded using a Leica TCS SP5 confocal microscope to capture gene expression patterns, with images processed and analyzed using Fiji and Inkscape for visualization by TW and JFO.

Timing and spatial scale: Whole-mount RNA Fluorescence in situ Hybridization (RNA-FISH) and Hybridization Chain Reaction (HCR-FISH) experiments, confocal microscopy, and image post-processing were performed from March 2023 to March 2025

Data exclusions: No data were excluded from the analyses.

Reproducibility: All attempts to repeat the experiment were successful.

Randomization: This study is primarily descriptive and focuses on qualitative gene expression analysis during the development of Spadella cephaloptera, rather than on statistical testing or experimental manipulations that would require randomization.

Blinding: The study involves qualitative analysis of gene expression in different developmental stages, there is no experimental condition or a comparison between groups (e.g., control vs. treatment) where observer bias could influence the results.

Did the study involve field work? ☒ Yes ☐ No

Field work, collection and transport

Field conditions: The intertidal zone in Roscoff, France was characterized by typical marine conditions during the sampling period (June), with temperatures ranging from 12°C to 16°C during the day, seawater salinity of ~35 ppt, and no major disturbances such as storms or unusually strong tides. The area is subject to pronounced tidal variability, and the timing of specimen collection was coordinated with low tide to ensure safe and effective access to the algae meadows where Spadella cephaloptera is commonly found.

Location: The sampling location is Roscoff, France (48°43'47.2"N 3°59'12.2"W). The water depth during the low tide in the intertidal zode ranges from 0.5 to 1 meter.

Access & import/export: The intertidal zone near the Roscoff Marine Station is easily accessible by foot, located just a short walk from the station. Researchers can reach the collection site by walking along the coastal path, which leads directly to the shoreline at low tide. The research conducted on Spadella cephaloptera was carried out in full compliance with the Access and Benefit-sharing Clearing-House (ABSCH) requirements, as evidenced by the Internationally Recognized Certificate of Compliance (IRCC) (ABSCH Unique ID: FR-265796-1; Date of Issue: November 08, 2023). Additionally, the research is in compliance with French ABS legislation, as attested by the French Certificate of Declaration (permit reference NOR:TREL2302365S/741; Date of Issue: November 08, 2023). Furthermore, a biological collection report was submitted, in line with the regulations for researchers hosted at the Station Biologique de Roscoff .

Disturbance: No disturbance caused during the field work.

Reporting for specific materials, systems and methods

We require information from authors about some types of materials, experimental systems and methods used in many studies. Here, indicate whether each material, system or method listed is relevant to your study. If you are not sure if a list item applies to your research, read the appropriate section before selecting a response.

Materials & experimental systems

n/a	Involved in the study
☐	☒ Antibodies
☒	☐ Eukaryotic cell lines
☒	☐ Palaeontology and archaeology
☐	☒ Animals and other organisms
☒	☐ Clinical data
☒	☐ Dual use research of concern
☒	☐ Plants

Methods

n/a	Involved in the study
☒	☐ ChIP-seq
☒	☐ Flow cytometry
☒	☐ MRI-based neuroimaging

Antibodies

Antibodies used: Roche Anti-Digoxigenin-AP, Fab-Fragments (Cat. No. 11093274910)

Validation: The Anti-Digoxigenin-Aklaline Phosphatase (AP) conjugate antibody used in this study was selected based on its established effectiveness for RNA-FISH applications. Its suitability for use in Spadella cephaloptera was supported by prior published studies (e.g., DOI: 10.1002/jez.b.23193 and 10.1186/s13064-024-00182-6) that successfully employed the same antibody and brand in this species and experimental context.

Animals and other research organisms

Policy information about studies involving animals; ARRIVE guidelines recommended for reporting animal research, and Sex and Gender in Research

Laboratory animals	The study did not involve laboratory animals.
Wild animals	Adult Spadella cephaloptera were collected from the intertidal zone in Roscoff, France, where they typically attach to surrounding algae. Collection was performed using a hand net and/or plankton net to comb through the algal meadows and capture specimens. During transport to the University of Vienna Aquatic Laboratory, animals were placed in plastic containers filled with fresh, natural seawater. To minimize temperature-induced mortality, the containers were surrounded by chilled water bags. In the laboratory, specimens were maintained in 50–70 liter aquaria containing a mixture of artificial and natural seawater, kept at 14°C and 35 ppt salinity, under a 16:8 light:dark photoperiod. At the end of the experiment, specimens were fixed in 4% paraformaldehyde in a buffer solution (0.1 M MOPS pH 7.4, 2 mM EGTA, 1 mM MgSO4, 2.5M NaCl) for downstream analyses, including in situ hybridization.
Reporting on sex	This information was not collected.
Field-collected samples	All samples collected from the field were were maintained in 50-70 liter aquaria with a blend of artificial and natural sea water kept at 14°C and 35 ppt, with a 16:8 light:dark photoperiod. At the end of the experiment, specimens were fixed in 4% paraformaldehyde in a buffer solution (0.1 M MOPS pH 7.4, 2 mM EGTA, 1 mM MgSO4, 2.5M NaCl) for downstream analyses, including in situ hybridization.
Ethics oversight	Ethical clearance was not applied for the Spadella cephaloptera used in this study, in accordance with the EU Directive 2010/63/EU. This species is not listed as a endangered, protected, or regulated species and not covered by CITES or national conservation laws that would restrict its collection for research. In addition, since chaetognaths are invertebrates not covered by institutional or governmental animal welfare regulations, no ethical approval or protocol review was required from any oversight organization.

Note that full information on the approval of the study protocol must also be provided in the manuscript.

Plants

Seed stocks	No plant specimens were used in this study.
Novel plant genotypes	No plant specimens were used in this study.
Authentication	No plant specimens were used in this study.
